# An integrated, tiered microplastic workflow, supporting rapid broadscale detection options

**DOI:** 10.1016/j.mex.2025.103536

**Published:** 2025-08-05

**Authors:** Samantha K Lynch, Colin L Johnson, Shivanesh Rao, Jaimie Loa-Kum-Cheung, Edwina L Foulsham, Alessandra L Suzzi, Lachlan Hill, Neil Doszpot, Rajitha Athukorala, Uthpala Pinto, Keegan Vickers, Maddison Carbery, Marina F.M. Santana

**Affiliations:** a Department of Climate Change, Energy, the Environment and Water, New South Wales, 2141, Australia; b ARC Industrial Transformation Training Centre in Data Analytics for Resources and Environments, Sydney, New South Wales, 2006, Australia; c School of Life and Environmental Sciences, The University of Sydney, New South Wales, 2006, Australia; d Australian Institute of Marine Science, Townsville, Queensland, 4810, Australia; e James Cook University, Townsville, Queensland, 4811, Australia; f AIMS@JCU, Townsville, Queensland, 4810, Australia

**Keywords:** Particles, Rapid count method, Nile red, FTIR, Density separation, Potassium hydroxide, Sodium chloride, Estuary, Manta net, Vacuum, Plastic, Monitoring

## Abstract

With growing concerns regarding microplastic pollution, there is an urgent need to improve understanding of their presence, distribution, and environmental impacts. This necessitates more coordinated and harmonised large-scale microplastic monitoring initiatives. However, such assessments are traditionally expensive, labour-intensive, and hindered by a lack of standardised sampling and analytical protocols, which impede rapid, yet accurate identification of microplastic sources and ecological risks. To improve environmental microplastic contamination estimates, this study proposes a rapid, cost-effective, and bulk-processing approach within a criteria-driven Tiered Microplastics Workflow (TMW). This approach enables the efficient quantification of microplastic contamination in estuarine surface waters, offering adaptable levels of analytical resolution, that is scalable for environmental monitoring. Key features of the TMW include:

• **Streamlined processing**: sieving, digestion, density separation, vacuum degassing, size-classed filtration, Nile Red staining, and automated fluorescent particle counts via a Python script, enabling 24 samples to be processed in five days.
• **Rapid Count Method:** Enabling microplastic identification in broadscale monitoring within a 20 % error margin. Script-based microplastic counts align with FTIR results (R² = 0.83).
• **Flexible resolution:** Sample processing can be paused and switched to other analytical methods while maintaining data comparability ensuring data harmonisation.

## Graphical abstract

**Figure:**
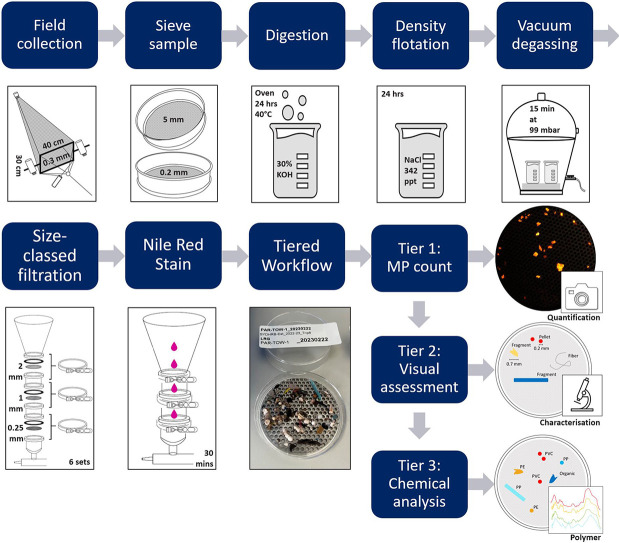

## Specifications table

**Table:** 

**Subject area**	Environmental Science
**More specific subject area**	Microplastic assessments of estuarine surface waters
**Name of your method**	The Rapid Count Method
**Name and reference of original method**	Nabi, I., A.-U.-R. Bacha, and L. Zhang, *A review on microplastics separation techniques from environmental media.* Journal of Cleaner Production, 2022. **337**. Wootton, N., et al., *Marine sampling field manual for microplastics*, in *Field Manuals to Monitor Australian Waters*, F.S. Przeslawski R, Editor. 2024, National Environmental Science Program. Kroon, F., et al., *A workflow for improving estimates of microplastic contamination in marine waters: A case study from North-Western Australia.* Environ Pollut, 2018. **238**: p. 26–38. Maes, T., et al., *A rapid-screening approach to detect and quantify microplastics based on fluorescent tagging with Nile Red.* Sci Rep, 2017. **7**: p. 44,501. Shruti, V.C., et al., *Analyzing microplastics with Nile Red: Emerging trends, challenges, and prospects.* J Hazard Mater, 2022. **423**(Pt B): p. 127,171. Schlawinsky, M., Santana, M.F., Motti, C.A., Martins, A.B., Thomas‐Hall, P., Miller, M.E., Lefèvre, C. and Kroon, F.J., 2022. *Improved microplastic processing from complex biological samples using a customized vacuum filtration apparatus*. Limnology and Oceanography: Methods, 20(9), pp.553–567. Santana, M.F.M., et al., *An assessment workflow to recover microplastics from complex biological matrices.* Mar Pollut Bull, 2022. **179**: p. 113,676. Service, N.E.D. *EMODnet micro-litter types*. 2023 25/07/2023 [cited 2025 17/02/2025].
**Resource availability**	NSW EPA Monitoring for Microplastics Video Rapid Microplastic Counter

## Background

Significant advancements have been made in microplastics research; however, globally, scientists have recognised that persistent challenges remain when working with environmental samples. Two primary challenges include the lack of reproducible, standardised sampling methods across different environments and the time-intensive nature of sample processing, both which affect the accuracy and consistency of results [[1], [2], [3]]. Currently, these challenges limit comprehensive spatial and temporal assessments, impeding the evaluation of policy effectiveness aimed at reducing microplastic contamination. While efficient and uncomplicated methods and workflows are crucial for large-scale environmental surveys, scientific rigor demands standardised methods to ensure data harmonisation that allows for cross-study comparisons [1,3,4].

Estuaries are key sites for microplastic monitoring due to their role as receiving waters for upstream and terrestrial contaminants [5,6]. Estuaries act as both sinks and temporary reservoirs for microplastics before their eventual transport to the open ocean [5]. However, a one-size-fits-all approach to marine surveys, such as that applied in ocean studies, is unlikely to be effective [7]. Existing marine-based sampling protocols are often difficult to directly apply or adapt to estuarine environments due to large environmental variations in waterway morphology, tidal influences, and complex surface water compositions that include suspended sediments, planktonic organisms, and organic debris.

To address the logistical and environmental challenges posed by estuarine samples, this study developed:

1. A standardised criteria-driven Tiered Microplastics Workflow (TMW) whereby sample processing and data acquisition can be tailored to the specific research question, while maintaining data comparability; and
2. A Rapid Count method to monitor microplastic abundance in estuarine surface waters.

**Fig. 1:**
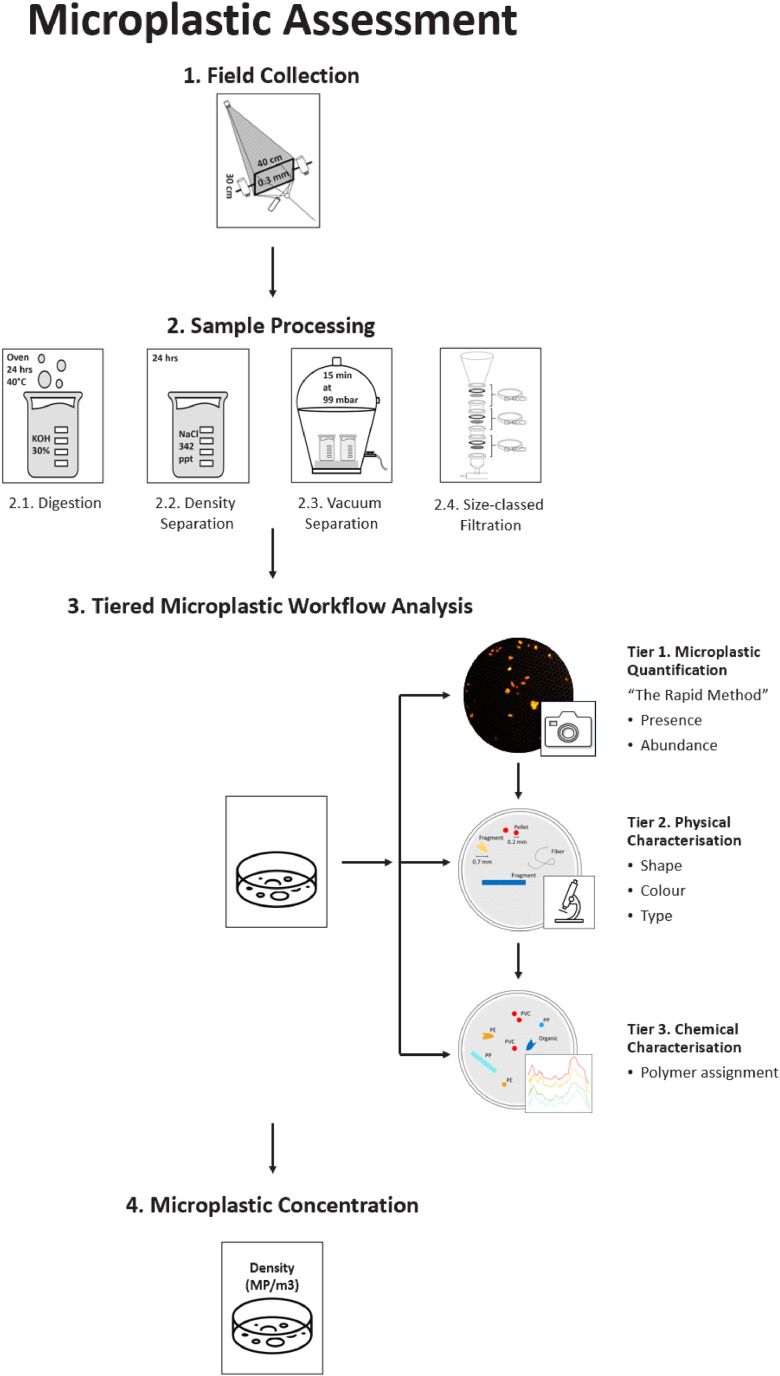
A standardised criteria-driven Tiered Microplastics Workflow (TMW).

Given the intricate composition of estuarine waters, four sample processing steps were first introduced to clarify samples for downstream analyses: 1) alkaline digestion using 30 % potassium hydroxide (KOH), which has high digestion efficiency for organic material but minimally affects plastics [[8], [9], [10]], 2) density separation using saturated sodium chloride (NaCl) solution, a versatile method for isolating buoyant microplastics from environmental water samples [1,11], 3) vacuum degassing, introduced to remove interstitial air pockets causing organic debris to sink while maintaining the buoyancy of synthetic plastic particles, and 4) size-classed filtration (Fig. 1).

The TMW integrates standardised photographic, and Fourier Transform Infrared Spectroscopy (FTIR) based polymer characterisation of clarified samples alongside robust data reporting guidelines, ensuring consistency and comparability. Designed for flexibility, it allows analysis to be halted or redirected at any stage while maintaining reliable results. Each step, detailed below as tiers, can be applied independently or in combination, depending on the nature of the sample and questions being asked (Fig. 1). Embedded within the criteria-driven TMW, Tier 1: the Rapid Count Method; a Nile Red (NR) staining protocol in combination with a Python numerical script, was specifically designed to analyse estuarine samples. The high microplastic loads in estuarine samples often require cost and time-effective bulk processing and analysis. The Rapid Count method, validated employing FTIR, enables rapid assessment, and when appropriate circumventing the need for detailed polymer characterisation. When more detailed assessments are required, Tier 1 can be skipped and samples can go from filter to FTIR (Tier 3).

## Method details

The purpose of this manuscript is to assist microplastic researchers in producing results tailored to the specific questions being asked, whilst still being comparable more broadly. Hence, the following protocol is organised as a standard operating procedure to facilitate reproduction. Subheadings denoted in Fig 1 have been utilised throughout to assist the reader. Validation of the TMW is presented on microplastics sized 0.25 to 5 mm, along with method limitations. Materials required are provided in the supplementary material. The supplementary material also provides more detailed information regarding QAQC.

## Field sample collection protocol

**Fig. 2:**
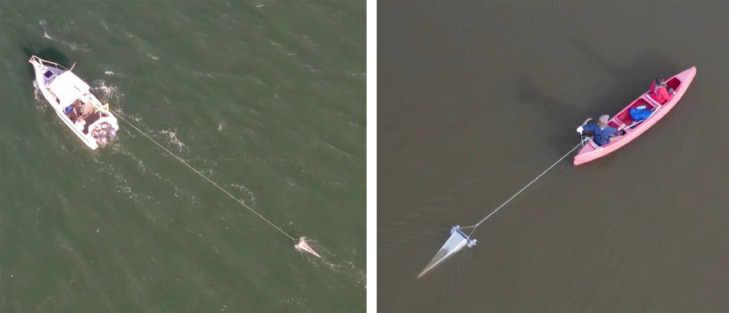
Images show the manta net being towed by a 6.4-m motorised vessel (left panel), and by a 3-m canoe rigged with a motor (right panel) on Parramatta River, NSW.

The field sample collection protocol was developed for adoption in estuarine environments. It was subsequently applied in 120 estuaries in New South Wales (NSW), SE Australia, (comprised of rivers, creeks, lakes, and lagoons), using various vessels (4.5-m vessel, 6.4-m vessel, and a 3-m canoe rigged with a motor) to sample surface waters for microplastics (Fig. 2; Table S1). Commonly, neuston nets with mesh size varying from 200 to 333 μm are used to sample plastics from rivers, oceans, and seas [1]. Here, a 300 μm mesh manta net capable of surveying the top 15 cm of the water column was specifically designed for sampling in smaller and shallower estuarine waterways, i.e., being towed behind a canoe (Fig. 2). If adopted in a larger motorised vessel, the net should be towed on the side of the vessel to avoid the collection of microplastics resuspended from the water column by the engine.

The field sample collection is performed as detailed below:

1. Once at the tow location, remove the manta net (herein ‘the net’) from its vinyl storage bag.
2. Attach the flowmeter to the two bottom bridles of the net – this positioning will minimise any damage to the mesh or introduction of contamination during deployment.
3. Connect the two top and two bottom bridles to a 15 m rope.
4. Tie off the rope to the rear cleat of the vessel.
5. Without the cod end attached, rinse the net by towing it for a period of 1 min.
6. Rinse the inside of the cod end 3 times with deionised and filtered water.
7. Screw the rinsed cod end onto the net.
8. Record the flowmeter measurement (revolution values) immediately before deploying the net, or tare if this function is available.
9. Deploy the net over the side of the vessel (Fig. S1), giving line until the net is clear from the motor, and is towing just outside the wake of the vessel (Fig. S2).
10. Whilst towing, ensure a 50 % height profile of the net within the water (i.e., 50 % of the net height remains above the surface and 50 % is submerged to filter the top 15 cm of the surface water).
11. Once the net is in correct position in the water, start the tow by recording the GPS coordinates.
12. Tow the net for a period of five minutes. Ensure the vessel maintains a straight course at a speed of approximately 4 knots.
13. After five minutes, place the vessel into neutral, record the GPS coordinates, and pull the net alongside the vessel.
14. Record the flowmeter measurement.
15. If present, remove large items (i.e., seagrass or other large organic or plastic debris) from the net and place into a clean Ziplock bag. Label the bag accordingly as this material might contain trapped microplastics and should be considered part of the cod end sample (record minimum information: location, tow number, date and time).
16. Rinse the contents of the net using the teabag method. This involves repeatedly dunking the net in the water and lifting out like a teabag, which rinses contents downward and into the cod end. Do not allow the opening of the net to be submerged in the water, otherwise there is a risk of losing trapped items.
17. Pour cod end material into the 500 mL sample jar pre-filled with filtered ethanol (100 mL 96 % filtered Ethanol, cellulose membrane <0.2 µm, or another filter of preference).
18. Repeat steps 16 and 17 twice, emptying the cod end contents into the same 500 mL sample jar, or if needed a second (or third, etc.) 500 mL sample jar.
19. Screw on the lid and seal the sample jar with duct tape.
20. Label the sealed sample jar accordingly (record minimum information: location, tow number, date and time).
21. Conduct a replicate tow at each location.
22. During field collection, collect field blanks to identify potential airborne or procedural contamination introduced during sampling. To do this, expose a blank sample by removing the lid of an empty sample jar filled with filtered deionised water to the field environment during sampling activities (whilst under tow).
23. While in the vessel, stow sample jars and bagged organic material on ice in an esky. On land, transfer the sample jar to a 4°C fridge and bagged items to a −18°C freezer until processing. Cold storage avoids mould growth and reduces bad odours that result from the decomposition of organic matter.

## Sample processing protocol

The sample processing method includes 30 % KOH digestion (outlined in section 2.1), saturated NaCl density separation (outlined in section 2.2), vacuum degassing (outlined in section 2.3) and size-class filtration (outlined in section 2.4) (Refer to Table S2 for list of materials required). Together, these steps ensure optimal sample clarification for downstream NR stain application. NR staining of samples, supported by automated image processing, is being used more frequently for facilitating the count of microplastics [12]. The advantages of NR staining are strong adsorption to plastics, high fluorescence intensity, short incubation time, and robust affinity for a wide range of polymers [9,[12], [13], [14]]. The disadvantages relate to over or underestimation of microplastics (discussed later) [15]. To fast-track processing and enhance analysis efficiency, the sample processing protocol was designed and tested against 24 complex estuarine surface water samples, the aim: to complete all 24 within one week. The processing protocols are performed as detailed below.

During processing, it is critical to have laboratory and procedural blanks to identify potential airborne or procedural contamination introduced during sampling. Whilst undertaking the laboratory processing and analysis (i.e., from sieving to FTIR), place a beaker with deionised and filtered water next to the technician with the lid removed as the laboratory blank, and process as outlined below. Additionally, fill a beaker with deionised and filtered water and process from start to finish as the procedural blank.

### Digestion protocol

1. Remove samples from the fridge/freezer and thaw to room temperature, including flied blank samples.
2. For each sample, including field, laboratory, and procedural blanks, triple rinse one 2 L glass beaker and one 150 mm D x 25 mm H petri dish (to be used as lid) with Reverse Osmosis (RO) water. Label beaker and lid with the same code as the sample jar.
3. Triple rinse the 5 mm and 0.2 mm sieves with RO water and stack the 5 mm sieve on top of the 0.2 mm one. Place the stack over a draining sink.
4. For each sample, pour sample jar contents over the stacked sieves (Fig. 3a), triple rinsing the sample jar with RO water into the sieve to ensure all sample contents are transferred (Fig. 3b).

**Fig. 3:**
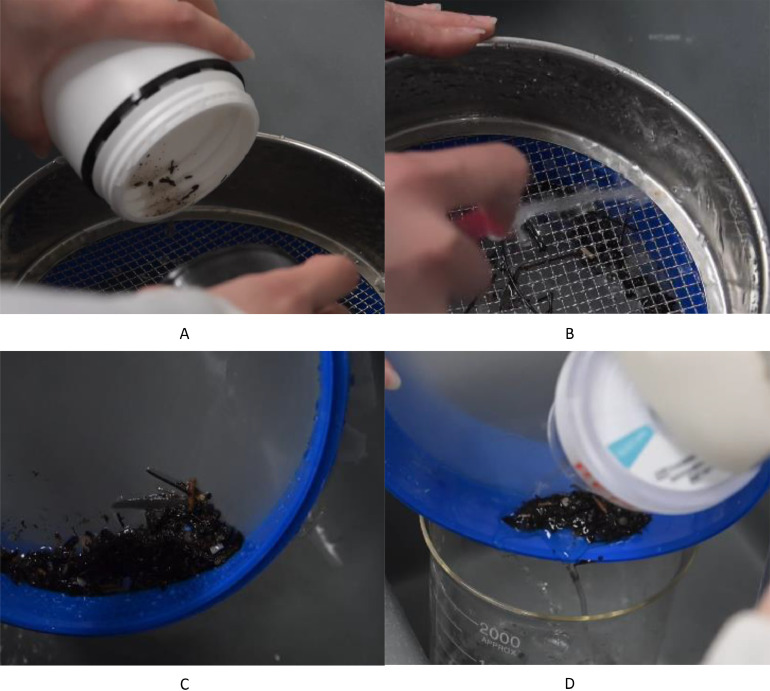
Images of the sieving process. A) the sample being poured over the stacked sieves; B) triple rinsing the sample jar contents over the sieve; C) digestate contents concentrated into the corner of the 0.2 mm sieve; and D) transfer of the digestate into a 2 L glass beaker (400 mL maximum).
5. If an associated Ziplock bag exists for the sample, pour the contents progressively through the stacked sieves. Triple rinse the bag over the sieve stack as per step 4.
6. Thoroughly rinse sample retentate trapped by the 5 mm sieve. This will ensure the retention of all material smaller than 5 mm but larger than 0.2 mm on the 0.2 mm sieve and facilitate sample clarification.
7. Using a squirt bottle filled with deionised and filtered water, concentrate the contents of the 0.2 mm sieve into the corner of the sieve (Fig. 3c) and then pour the particulate residue into the labelled 2 L glass beaker (Fig. 3d). Do not add more than 400 mL of deionised and filtered water into the glass beaker as this measurement is needed to calculate 30 % KOH. If less than 400 mL is needed to do the transfer, make up the final volume in the glass beaker to 400 mL with additional deionised and filtered water.
8. Samples high in organic matter (i.e., > 400 mL) will need to be split across two or more glass beakers, each labelled with the sample code followed by A or B, etc.
9. Under a fume hood, and wearing the appropriate PPE (gloves, lab coat and eye protection), slowly add 214 g (pre-weighed) of KOH pellets to the 2 L beaker. Stir the mixture of KOH, deionised and filtered water and sample with a glass stirring rod until KOH pellets are fully dissolved.
10. Using 100 mL of deionised and filtered water, rinse the glass stirring rod and the side of the glass beaker with a squeeze bottle, ensuring all sample contents are submerged in the solution. Please note KOH dissolution is an exothermic reaction and the final addition of this 100 mL of deionised and filtered water will return the solution to room temperature. The total weight of KOH (214 g) and final volume of deionised and filtered (500 mL) will result in a 30 % KOH solution.
11. Place the lid on top of the 2 L glass beaker to avoid extraneous lab-derived microplastic contamination.
12. Place the beaker in a 40°C oven for 24-hours.
13. After 24-hours, remove the sealed beaker from the oven and allow it cool down to room temperature.
14. Place a 20 L waste bucket and a 0.2 mm sieve (pre-cleaned as per step 3) in a fume hood.
15. Wearing the appropriate PPE (gloves, lab coat and eye protection), pour the cooled digestate in the 2 L glass beaker over the 0.2 mm sieve, allowing the KOH solution to drain into the waste bucket.
16. Triple rinse the 0.2 mm sieve with deionised and filtered water to concentrate digestate contents into the corner of the sieve (Fig. 3c).
17. Neutralise the waste KOH with acetic acid (approx. 200 mL per sample). Use pH strips to test when neutralisation is achieved and then dispose of the waste according to Safety Data Sheet requirements.

### Density separation protocol

1. Using a squeeze bottle filled with pre-filtered 342 g/L NaCl solution, transfer the digestate into a pre-cleaned 400 mL glass beaker (clean beaker as per step 2 of Digestion protocol).
2. Add additional NaCl solution, as needed, to give a final volume of 300 mL and cover the 400 mL beaker with a pre-cleaned petri dish lid.
3. Sonicate the sample for 15 min (number of samples sonicated at one time is dictated by the size of the sonicator bath).

### Vacuum separation protocol

1. After sonication, place the 400 mL beaker into a vacuum desiccator chamber (Fig. 4) for 15 min at 99 mbar (for this size vacuum desiccator 3 batches can be degassed).

**Fig. 4:**
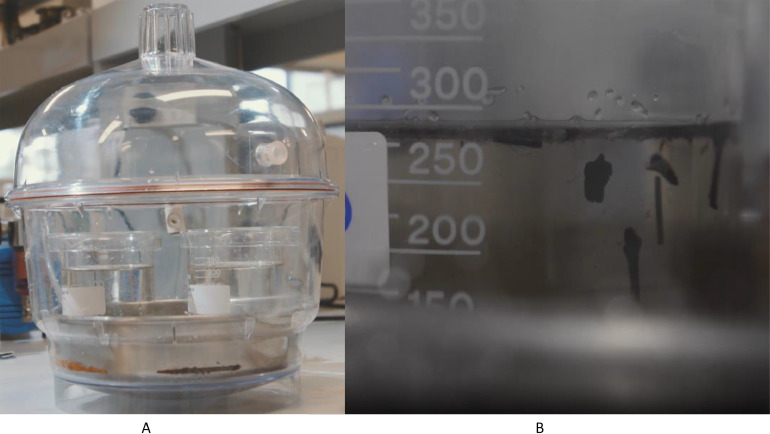
Vacuum degassing using a vacuum desiccator chamber (image A). The desiccator chamber is attached to a vacuum pump. Once under vacuum the desiccator chamber will seal. The sample is degassed for 15 min (99 mbar) to remove interstitial air pockets from the particulates, i.e., driftwood (shown in image B), causing them to sink while synthetic particles retain their buoyancy (Adapted from Kunz & Siña, 2021; see from 7:00 onwards [16]).
2. By visual, if, after 15-minutes, organic matter contents are still on the surface, extend the degassing for a further 15 min.
3. Isolate the chamber and turn off the vacuum. Slowly bleed the chamber so that the samples are not disturbed.
4. Leave the degassed sample to settle overnight in the chamber for enhanced separation of microplastics from the organic matter.

### Size classed filtration protocol

**Fig. 5:**
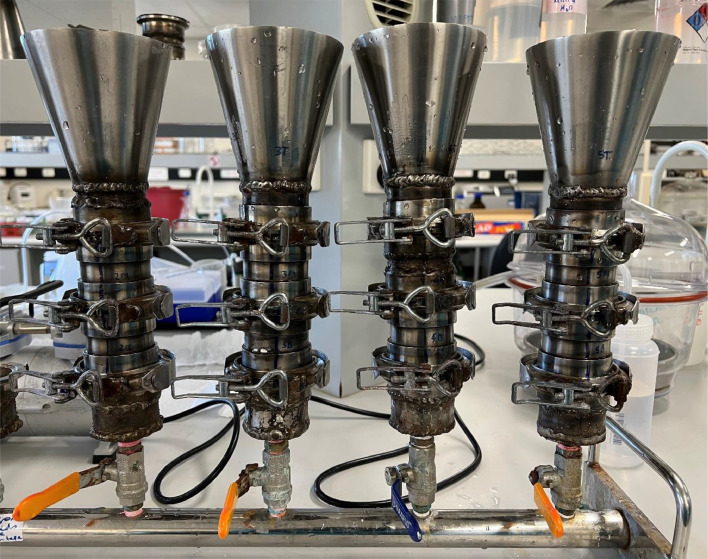
Size classed filtration system adapted from Schlawinsky, Santana [17]. The system is comprised of a series of stainless-steel expanders that act as stacked sieves, attached to a vacuum manifold. The use of a vacuum manifold allows for multiple stacks to be used simultaneously (four stacks shown here), whilst providing a stable base. The ball valves allow the stacks to be individually drained when open, or to soak in Nile Red (NR) stain when closed. Stainless steel materials were used to reduce corrosion and the potential of extraneous contamination of the samples.

1. Rinse the size classed filtration system (Fig. 5; adapted from Schlawinsky et al. 2022 [17]. Here, the filtration system consists of six inline filtration stacks and can filter 6 samples simultaneously) thoroughly with deionised and filtered water and assemble it (Fig. 6; refer to Schlawinsky et al. 2022 [17] for operational guidelines). Place the smallest filter mesh size on the bottom of the filter stack (0.25 mm), then the medium sized filter disc (1 mm), and lastly the large filter disc (2 mm).

**Fig. 6:**
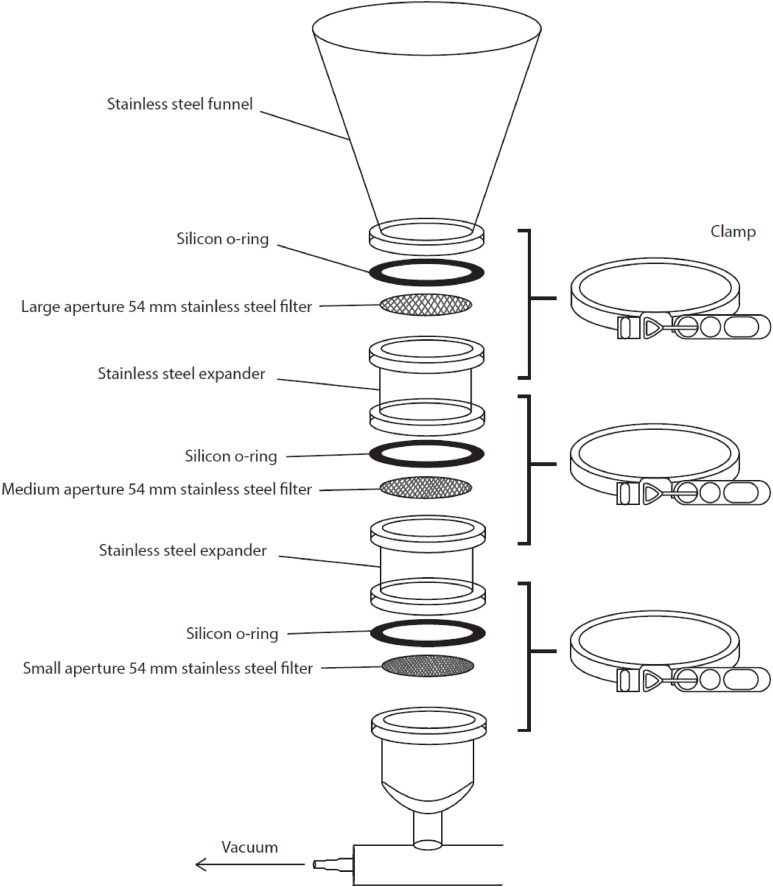
Size classed filtration system adapted from Schlawinsky, Santana [17]. Each filtration stack consists of a stainless-steel funnel, a series of three self-aligning flanges (stainless steel expanders) to accommodate the stainless-steel filter discs, and a ball valve drain attached to a vacuum manifold. Self-aligning flanges are sealed with a silicone O-ring and secured with v-band clamps, that were modified to be “quick release” with an over centre latch. The silicon-O-ring is added to the in-house contamination reference library.
2. Turn on the vacuum pump and make sure the system is sealed, and the vacuum is working properly.
3. Decant approx. 100 mL of the supernatant from the settled sample into one of the filtration stacks (Fig. 7a).

**Fig. 7:**
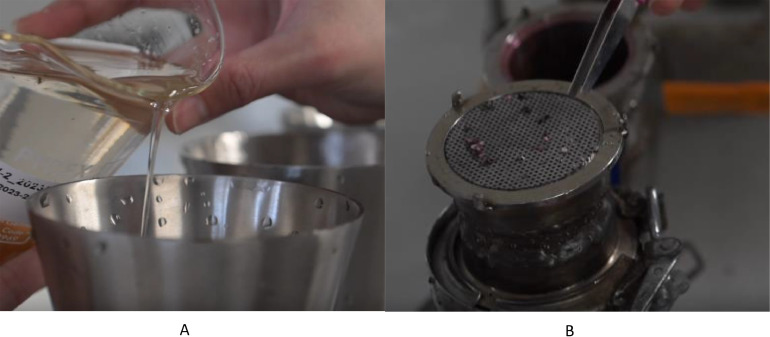
Images of sample filtration using the size classed filtration system, A) demonstrating the decanting of the sample into the funnel, and B) particulates trapped on one of the stainless-steel filter discs.
4. Rinse the inside walls of the 400 mL glass beaker with NaCl solution to wash particulates back into the solution.
5. Repeat steps 3 and 4 three times. After this, the remaining 100 mL of brine solution and deposited organic material in the glass beaker can be discarded.
6. To fully remove traces of the NaCl, rinse each filtration stack with 2 L of deionised and filtered water. For the last 200 mL, close the ball valve and soak the filter stack for 5 min, after which open the ball valve and allow the water to fully drain.

Steps 7 to 11 for NR staining should be conducted only if the sample was deemed complex thereby *requiring Tier 1 analysis* (Fig. 1)*. If the sample is considered simple and NR staining is not required proceed to Tier 2.*

1. For optimal performance, prepare the NR stain on the day of use. Add 1 mg of NR stain to 1 mL of acetone . NR solution is light sensitive. Keep the NR stain solution in an amber jar (or a bottle covered in foil) and shake well prior to use [12].
2. Add 140 mL of deionised and filtered water and 1.5 mL of the NR-acetone solution to a 200 mL glass measuring cylinder.
3. With the ball valve of the filtration stacks closed, transfer the combined 141.5 mL aqueous NR-acetone solution to each stack (for 6 samples 849 mL is required). Rinse the side of the funnel with deionised and filtered water (maximum 10 mL each stack), ensuring all sample contents are in direct contact with the staining solution.
4. Leave the samples steeping for 30 min; considered optimal timing [12,14].
5. After 30 min, open the ball valve, and, as the NR-acetone solution drains, rinse each filtration stack with 2 L of deionised and filtered water.
6. Dismantle the filtration stacks, ensuring the side walls are well rinsed and each component is rinsed thoroughly to avoid loss of microplastics adhering to the system’s walls.
7. Once the filter discs are exposed (Fig. 7b), using forceps, carefully transfer the filter discs into labelled petri dishes and seal with a petri dish lid. Leave loaded filter discs to dry covered at room temperature for a minimum of 2 h and a maximum of 24 h or until the sample is dry - wet microplastic increases reflection when photographing [12].
8. Ensure all field, laboratory, and procedural blanks are processed using the same protocols listed above (digestion, density separation, vacuum separation, and filtration), and analysed to the desired tier following the same protocol as detailed below.

## Tiered microplastics workflow (TMW)

**Fig. 8:**
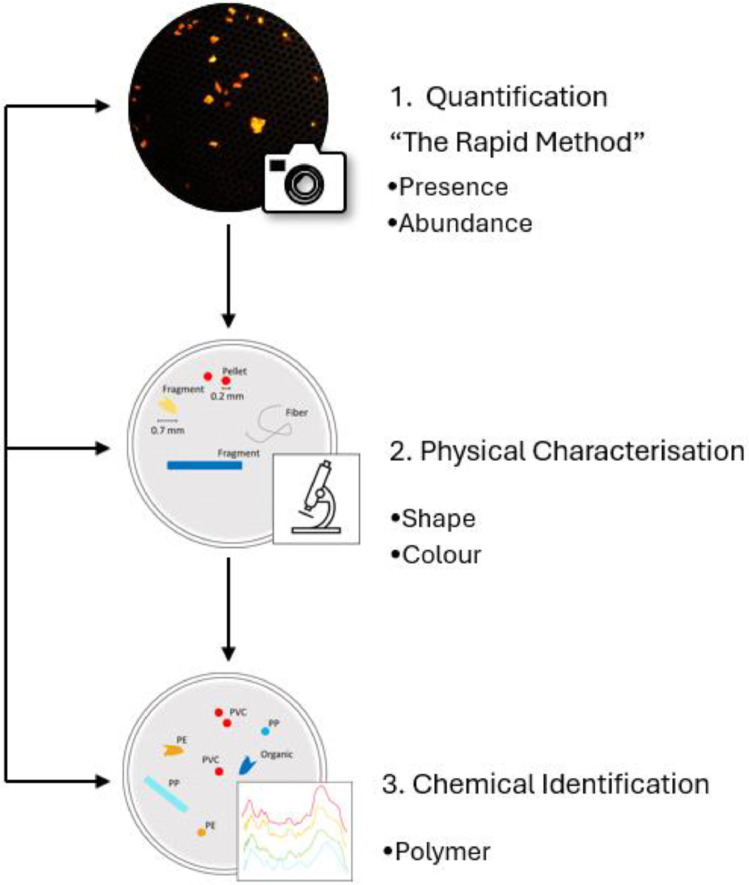
The criteria-driven Tiered Microplastics Workflow.

Globally, various analytical techniques have been developed to quantify microplastic contamination in the environment, including visual techniques such as stereomicroscopy and fluorescence-guided identification (i.e., NR), spectroscopic techniques such as FTIR and Raman, and spectrometric techniques such as pyrolysis-gas chromatography coupled with mass spectrometry (Pyr-GC/MS) [[18], [19]]. Established here, is a criteria-driven TMW designed to generate precise levels of information, from physical counts through to chemical polymer properties, to answer specific research questions within set timeframes, and often under resource constraints (Fig. 8). Within the TMW each level of information functions as a distinct tier with an opt in / opt out gated structure:

• Tier 1: provides low-resolution presence data (i.e., counts based on visual identification) which is translated into abundance data (i.e., microplastic counts per volume of water sampled),
• Tier 2: describes physical characteristics (i.e., size, shape, texture, defining morphological features, and colour) [[20], [21]] using a rapid option such as photographs and a higher resolution option stereomicroscopy; and
• Tier 3: establishes the chemical composition and confirms microplastic assignments [18].

### Tier 1: the rapid count method

**Fig. 9:**
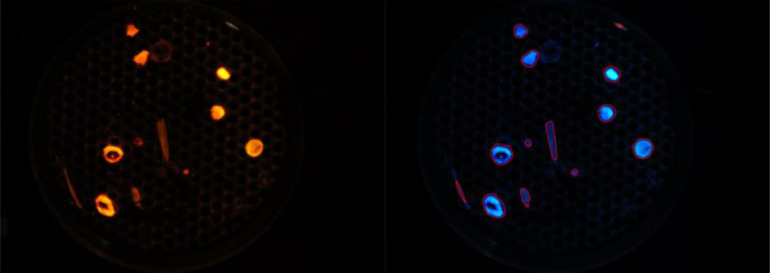
The ‘Stat-on-pixels’ Python script reads the RAW image (.jpg) file (left panel) and outlines and counts the perimeter of each fluorescent particle (right panel).

NR staining and low-resolution photography under blue light was utilised to rapidly assess microplastic contamination in estuarine environments and establish abundance data. A Python-language based counting script, ‘Stat-on-pixels’, available on GitHub, was created to automate the counting of fluorescent particles (Fig. 9). Stat-on-pixels works by splitting the image into the respective RGB colour bands, then applies a gaussian blur with a user-defined kernel size, and segments that image based on a spatially adaptive threshold value. The contours of the objects in the image are then extracted and filtered based on target size to produce a count governed by the selected spatial adaptive threshold(s). The materials required for the Rapid Count Method are listed in Table S3. The Rapid Count method is performed as detailed below:

1. In a dark room, attach a digital camera to the retort stand and affix the orange filter to the lens.
2. To ensure a consistent area of image capture, mount the petri dish containing the sample filter disc onto a permanent fixture (here, two fixed position pins were used, to optimise camera positioning; Fig. 10). Note this assembly will need to be tailored to the dark room and conditions of each laboratory, and the camera.

**Fig. 10:**
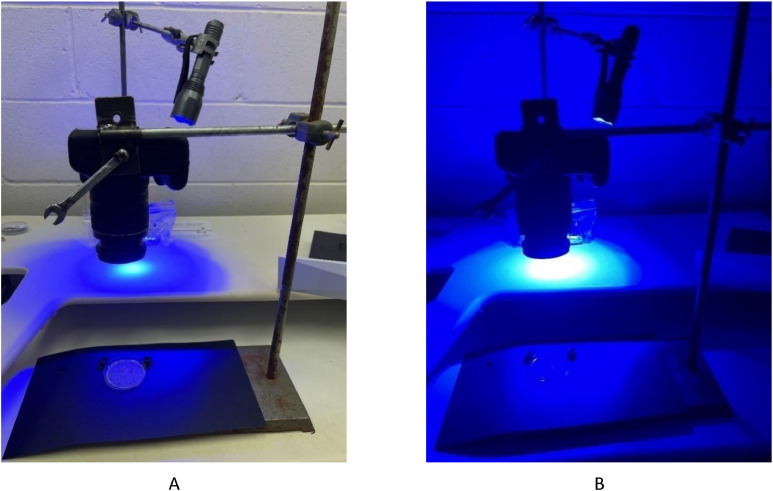
Dark room assembly showing A) the blue light torch and digital camera mounted on retort stands and the camera positioned perpendicular to the petri dish containing the filter disc (Camera lens is positioned 18 cm and torch 50 cm from the Petri dish), and B) during image capture under blue light.
3. Next, optimise the position of the camera lens such that it sits perpendicular to the filter disc and captures clear images of the filter disc surface (Fig. 10A; here the optimal distance was 18 cm).
4. On a second retort stand, mount the blue light source. The angle of the blue light source needs to be adjustable and optimised during photography to minimise reflections on the edges of the petri dish. Here, the optimal position of the blue light torch was upright and 50 cm from the filter disc (Fig. 10B).
5. Once optimised, position the sample filter disc using the fixed position pins.
6. Under normal light conditions, capture an image of the sample label to associate successive photographs with their respective sample. Ensure all photographs of samples are captured with a resolution of 6000 by 4000 dpi in raw image format (.jpg).
7. Turn off the light. Wearing the appropriate PPE (UV-blockage googles), turn on the blue light source.
8. Capture multiple photographs of the sample while manoeuvring the blue light source by rotating the torch through 180-degrees, starting at 09:00. This provides a series of images from which to select the one(s) giving optimal illumination of the microplastics with minimal interference from reflections.
9. Turn the blue light source off and remove googles.
10. Once photographs have been taken, download all images onto the hard drive, i.e., using an SD card reader. Then select the best image (optimal illumination with minimal interference) for each sample.
11. Referring to the first image taken of the sample, use the label name to save the best image into a folder called, for example, ‘Microplastic Script Count’.
12. Open the freeware on GitHub.
13. On the GitHub page open the folder ‘Microplastic Script Count’ and press enter to run the script. When completed, a .CSV file will be generated within the selected folder listing the name of each image and associated counts.

### Tier 2: physical characterisation

Here, the physical characteristics of microplastics were determined by visual inspection (Table S4 lists the materials required). For a rapid assessment, microplastics > 2 mm in size were characterised from photographs under white light, those < 2 mm in size were analysed using stereomicroscopy and high-resolution photographs. Physical characterisation (refer to Table S5 and S6) is performed as detailed below:

1. When photographing for the Rapid Count Method (Tier 1; NR and fluorescence captured by photography), or for physical characterisation (Tier 2; low-resolution photography under white light), attach the digital camera and the white light source to the retort stand and optimise its position relative to the sample filter disc (Fig. S3). Secure using the fixed position pins. Here, optimal positioning was established with the light at a 45-degree angle to the petri dish and 10 cm away.
2. Secure the glass petri dish and sample filter disc under the digital camera using the fixed position pins.
3. Capture an image of the label, and proceed to photograph the sample filter disc, as per Tier 1 step 6, producing images as shown in Fig. 11.

**Fig. 11:**
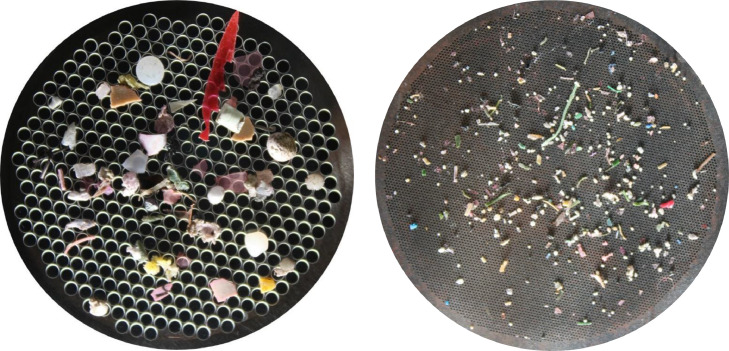
Example of low-resolution photographs of sample filter discs. Left panel shows microplastics on a 54 mm D, 2 mm aperture size filter disc. Right panel shows microplastics on a 54 mm D, 0.25 mm aperture size filter disc.
4. Save photographs of the sample filter disc, as per Tier 1 step 10.
5. For a rapid assessment, open the image in the program GIMP (GNU Image Manipulation Program). Create a layer for each microplastic morphology (Table S6). In each layer, select each individual microplastic matching the associated morphology, by pointing and clicking on the photograph. For example, on the foam layer, click all microplastics which appear to be foam.
6. Count the number of microplastics per morphology and enter that count against the sample code in a spreadsheet (i.e., excel).
7. For a more detailed analysis, open the image in ImageJ (freeware) and using the ‘multi-point ‘tool select each individual microplastic by pointing and clicking on the photograph. Assign particle numbers in a Z motion, i.e., row-by-row. Each item will now have a unique number. Save the image file as a .tiff file.
8. In a spreadsheet (i.e., excel), create a row for each unique particle as numbered in the ImageJ (.tiff) file. Document the observed physical characteristics of each unique particle including colour, morphology, and any other defining features (refer to Table S5 and S6) [[20], [21]].
9. Position the sample filter disc on the stereomicroscope and view, under magnification, each unique particle. Verify physical characteristics established in step 8 and, if necessary, record additional descriptors.
10. For steps 8 and 9, assign the colour based on the closest match to the colours depicted in Table S5 and morphology as described in Table S6.

### Tier 3: polymer identification

The only definitive way to confirm items as microplastics is by determining their chemical composition. Here, FTIR was used to chemically profile those items identified via Tier 1 or Tier 2 as putative microplastics, generating a spectrum which is then compared against a spectral reference library [18]. Here, the chemical composition of particles was determined using the Nicolet iS50 FTIR Spectrometer and the NICODOM Polymers All Package reference library. Table S7 lists the materials needed. Polymer identification is performed as detailed below:

1. Using forceps, transfer each individual numbered particle (see Tier 2 step 8) from the sample filter disc to the FTIR analysis platform (here attenuated total reflection (ATR) was used).
2. Using FTIR, measure the chemical signature of the particle. Here, the FTIR parameters included: 32 scans, wavelength between 1000 and 4000 cm^-1^, with 4 cm^-1^ of resolution operating in ATR mode.
3. Screen the spectrum against the spectral reference library. Here, OMNIC software was used to screen against the NICODOM Polymer All Package reference library and identify the most representative match. As several matches may be returned, always consider the top 10 spectral matches and assess whether the closest match is appropriate given the observed peaks. At this point, information pertaining to the morphology of the particle may provide guidance, e.g., artificial grass is predominately made of polypropylene, polyethylene, or a combination of the two, so a match to one of these polymers is expected for this morphology.
4. Only putative microplastics matching with synthetic polymers in the NICODOM spectral library, at a hit rate of at least 70 %, were considered for final chemical characterisation and microplastic counts [18].

## Concentration calculations

Variability in sampling methodologies and inconsistent data reporting have limited the effectiveness of current datasets and hindered broadscale and long-term comparisons [2,3]. However, for any microplastic surface water sample collected, if volume can be measured or calculated, the microplastics concentration can be determined and samples directly compared. Concentrations for each method were calculated by:

1. Counting the number of particles observed in each surface water sample.
2. Calculating the volume of surface water surveyed based on the flowmeter distance recorded (cm) and the net interface (40 × 15 cm); and
3. Per sample, dividing the total number of microplastics counted by the volume of water collected to give the total number of microplastics per cubic meter of water (MP/m^3^).

## Quality assurance and quality control (QA/QC)

Minimising microplastic contamination is essential during sample collection and processing [18,22]. Controls used here to minimise microplastic contamination included covering samples, triple rinsing equipment, and coloured lab coats. Further details on minimising contamination can be found in Table S8. It is also essential that blank controls are implemented during sample collection and processing to detect extraneous contamination [3] and ensure data integrity. A description of the type of blank controls applied here are listed in Table S9.

## Method validation

Each method described above has been optimised and cross validated. Selection of the optimal sample processing (i.e., digestion, density separation, filtration) protocol was based on the assessment of multiple different processing techniques, and combinations thereof, to produce clarified samples and high recovery rates of microplastics from estuarine surface waters, with capacity for bulk downstream analyses. Assessment was conducted with spike-recovery test samples (comparing the number of microplastics introduced to the number of microplastics recovered after digestion, density separation and filtration) and a subset of environmental samples (comparing clarification efficiency and number of microplastics detected across samples). The Python language-based script was validated by comparing known microplastic counts to script counts, as well as comparing script counts to FTIR counts. Physical characterisation of microplastics established by NR and fluorescence low-resolution photography was validated by comparing results from low-resolution photography to stereomicroscopy. Finally, the potential impact of the sample processing protocol on the chemical integrity and thus the accurate polymer identification of microplastics, was evaluated by comparing the FTIR spectra of spiked (i.e., processed/treated) particles with their original (pre-treatment) spectra and corresponding reference library spectra. Validation is described below:

### Sample processing protocol

**Fig. 12:**
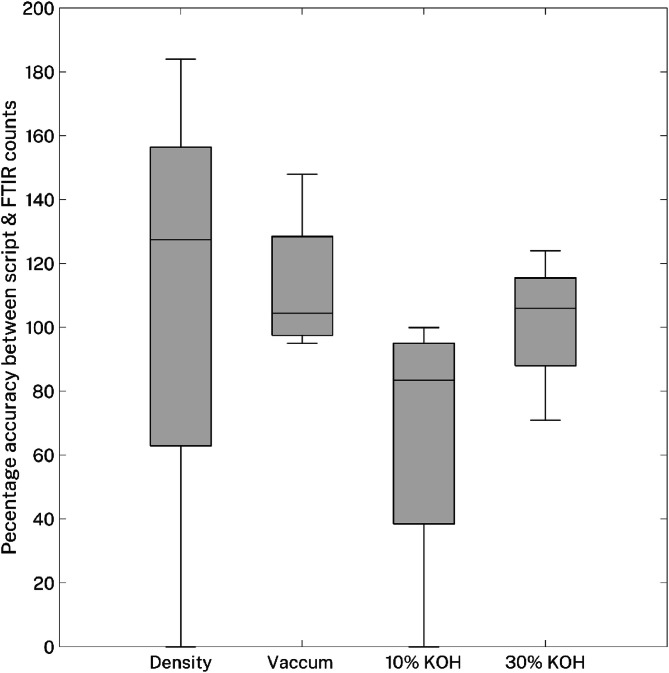
Box and whiskers plot displaying the percentage accuracy range of the Python script (Tier 1) counts when compared to the FTIR (Tier 3) counts under different treatments (density separation, vacuum degassing, 10 % KOH, and 30 % KOH). 100 % demonstrates that the script accurately counted 100 % of the microplastics confirmed by FTIR. >100 % indicates an overestimate of microplastics and <100 % an underestimate.

Optimisation of the sample processing protocol for high-load (Tier 1) estuarine surface water samples was performed using a subset of four replicate tow samples collected from four separate locations in Sydney Harbour (Lane Cove River, Middle Harbour, Parramatta River, and the main basin of Port Jackson) (*n* = 16 samples in total). One tow replicate from each location was processed by 1) density separation only [23], 2) density separation under vacuum, 3) digestion in 10 % KOH [10], or 4) in 30 % KOH. All 16 processed samples were filtered, NR stained and photographed as per the Rapid Count method. Putative microplastics (i.e., items that fluoresced) were then physically characterised by low-resolution photography and stereomicroscopy (Tier 2) and chemically analysed by FTIR (Nicolet iS50) to confirm their assignment (Tier 3). The Tier 1 script counts and Tier 3 FTIR counts were compared by 2-way ANOVA. Sample processing treatment had no significant impact (df=2, *F* = 0.25, *p* > 0.75) on microplastic counts. Still, undigested samples treated by density separation alone, as well as those subjected to density separation with vacuum degassing, retained substantial amounts of the sample matrix, including naturally derived (i.e., non-microplastic) fluorescent particles and natural debris, both of which impacted the final counts. The vacuum degassing caused a portion of the natural debris to sink, resulting in greater sample clarification and less variability compared to density separation alone. The two digestion treatments, in the absence of a density separation step, resulted in low sample clarification, with a higher retention of sample digestate. This impeded visual assessment and prevented an accurate count of particles, leading to higher rate of undercounting (Fig. 12). However, digestion with 30 % KOH yielded the least variability between replicates and was the most effective at removing a substantial amount of organic matter. Similarly, the vacuum degassing step improved sample clarity by causing fibrous and woody debris to sink. Given this, and the need for count accuracy, a processing protocol that combines 30 % KOH digestion with density separation and degassing under vacuum is recommended to achieve the greatest sample clarification.

### Microplastic recovery rate

To validate the sample processing protocol, microplastic recovery rates were determined using six spike-recovery test samples. Seven different microplastic polymers (PS, PE, PP, PVC, LDPE, PET, EPDM, abbreviations defined in Table 1) of various sizes (5 to 0.25 mm), colours (blue, yellow, green, grey, red, and white) and morphologies (fragment, filament, and foam) were used to prepare the spike-recovery samples, chosen as representatives of the most common plastics found in environmental samples [24].

**Table 1:** Common plastics polymers, their abbreviations, and densities*.

Plastic type	Abbreviation	Colour	Shape	Density (g/cm³)
Polyethylene Terephthalate	PET	fluorescent yellow	irregular fragment	1.38
Polyethylene	PE	yellow	irregular fragment	0.94 – 0.97
Polyvinyl Chloride	PVC	grey	irregular fragment	1.1 – 1.4
Low-Density Polyethylene	LDPE	green	elongated fragment	0.91 – 0.94
Polypropylene	PP	blue	irregular fragment	0.86 – 0.92
Polystyrene	PS	white	round foam	0.96 – 1.05
Ethylene: Polypropylene	EP:DM	red	filament	0.90 – 0.97

⁎ Density of surface ocean water is 1.03 g/cm³.

Validation by spike-recovery is performed as detailed below:

1. Six scintillation vials, each containing the seven microplastic polymers in various ratios (to mimic sample heterogeneity; Table S10), were prepared by the Australian Institute of Marine Science – this allowed for blind assessment. The contents of the scintillation vials were added to the treatment beakers (*n* = 3 blank deionised and filtered water controls and *n* = 3 positive controls – prepared using residual organic material from previously processed samples, i.e., having had all environmental plastics removed).
2. Spiked samples were processed using the optimised sample processing protocol. After each processing step, a visual count of the microplastics was performed and recorded (i.e., post sieving, digestion, density separation, and filtration).
3. Final recovery rates were calculated by dividing the total number of microplastics counted post filtration by the starting number of microplastics in the vial and then multiplied by 100 to calculate the percentage of microplastics recovered.
4. The filter discs were then photographed under blue light and counted by the script as per the Rapid Count method (Tier 1).
5. The script accuracy was calculated by dividing the total number of script counts by the starting number of microplastics in the vial and then multiplied by 100 to calculate the percentage of microplastics counted by the script.

**Fig. 13:**
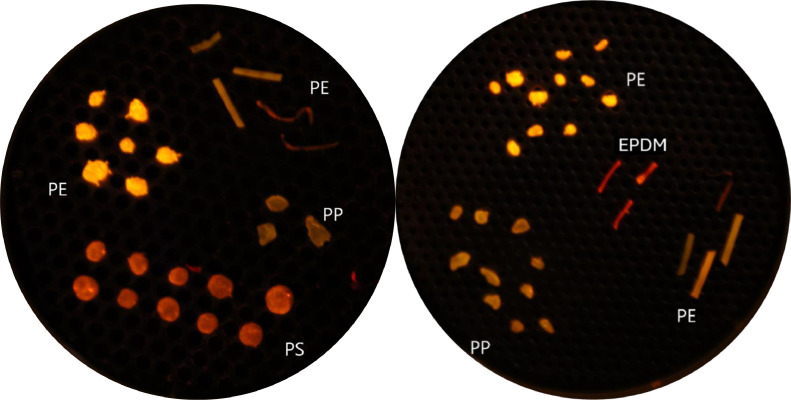
Plastic polymers stained with Nile-Red. Low-resolution photographs were taken in a dark room, under blue light and using an orange filter lens. Each polymer exhibits a distinct fluorescence emission spectrum and hence is a different shade. Refer to Table 1 for polymer abbreviations.

The average microplastic recovery rate across all six test samples was 78 %. Due to their higher densities, PVC and PET microplastics sunk to the bottom of the beaker in all samples and, therefore, were not always filtered with the supernatant (Table 1). Given estuarine surface waters would not typically contain PVC and PET they were removed from the final counts, which resulted in an average microplastic recovery rate of 99 % (Table S10). Of the 431 (PE, LDPE, PP, PS, EPDM) punitive microplastics included in the data analysis, 2 were lost during digestion and 3 during filtration. The script count accuracy ranged from 99 – 103 %, with a mean accuracy of 100 % (Table S10). Most microplastics in the test samples adsorbed the NR stain (Fig. 13), however, the green PE (i.e., artificial grass), and the white foam (PS) exhibited weaker fluorescence compared to the others and this occasionally hindered script-based microplastic detection.

### Script count validation

Validation of the Rapid Count method was achieved by comparative analysis of retained items on 35 filters. A subset of environmental samples were processed as per the optimised sample processing protocol, and filtered onto different size-classed filter discs: large (LRG, 2 mm, capturing items 5 to 2 mm in size; *n* = 10), medium (MED, 1 mm, capturing items 2 to 1 mm in size; *n* = 10) and small (SML, 0.25 mm, capturing items 1 to 0.25 mm in size; *n* = 15). Each filter disc was analysed using the Rapid Count method (Tier 1), and physically characterised (i.e., stereomicroscopy (Leica m80); Tier 2) and chemically (FTIR; Tier 3) characterised. To evaluate the reliability of script counts across different microplastic size ranges, final estimates of microplastic counts from both Tier 1 and 3 were compared and R² values used to assess the strength of the linear relationship between methods and establish an accuracy threshold (percent of correctly identified fluorescing items verified by FTIR). Overestimations were determined based on the number of non-plastic FTIR assignments.

**Fig. 14:**
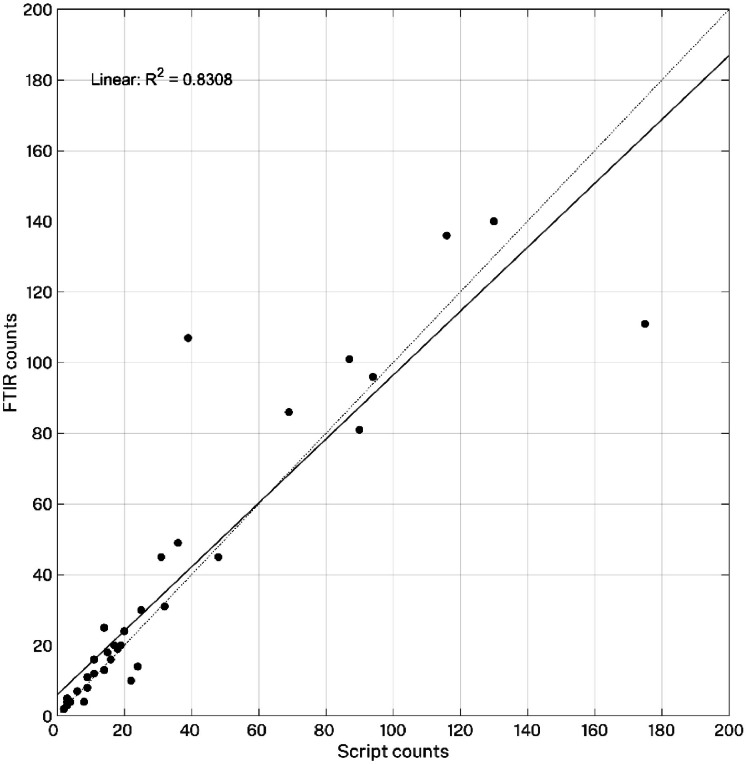
Scatter plot displaying the relationship between microplastic counts from the ‘Stat-on-Pixels’ python script and the FTIR spectral confirmation. R^2^ = 0.83.

Overall, NR staining combined with script counts reliably estimated microplastic abundance, showing a strong positive linear correlation with the FTIR method (R² = 0.83; Fig. 14). With the FTIR counts as the reference, the Rapid Count method was deemed reliable when script counts ranged between 80 % and 120 % of the FTIR counts. This 20 % margin of error is in accordance with previous studies [[12], [13], [14], [15],^25^], with size of the microplastics found to be a contributing factor. The Rapid Count method accurately counted more than 80 % of all items of all sizes 81 % of the time and overestimated counts by over 120 % only 8 % of the time. However, the performance of the script decreased as microplastic size decreased (Fig. S4), corroborating previously reported limitations of NR staining for microplastic detection [15]. Eighty percent of items >2 mm were accurately identified as microplastics 80 % of the time. Similarly, more than 80 % of items between 1 – 2 mm were accurately counted by the script count 87 % of the time, with overestimations occurring 13 % of the time. The smaller sized microplastics (1 – 0.25 mm) had the greatest error range, with the script counts accurately identifying more than 80 % of the small items 76 % of the time while overestimations increased and occurred 12 % of the time. Despite this variability, the script performs well when fewer than 100 microplastics are present per SML filter.

By comparing NR fluorescence low-resolution photographs and stereomicroscopy high-resolution photographs, it was concluded that overestimates were mainly caused by keratin (e.g., zooplankton carapace) and cellulosic items adsorbing the NR stain, and consequently fluorescing giving false ‘plastic’ positives. Underestimates were associated with microplastic size (< 0.25 mm), weathered polymers (e.g., PS monocarboxylic terminated), dark pigmented items (e.g., black coloured nurdles and dark green artificial grass fragments) and fine fibres (due to their low total surface area) – all impeding dye absorption - or when microplastics clump and overlap on the filter (i.e., script counts a clump as a single item).

### Characterising microplastics using low-resolution photography

To validate the use of low-resolution photographs for the rapid identification of larger sized microplastics from large and complex sample sets, counts from low-resolution photographs of ten LRG (5–2 mm) filter discs (analysed blind by three separate researchers (SL, JL, JM) following Tier 2 protocol steps 1 – 6), were compared to those obtained from stereomicroscopy for the same ten filter discs (analysed by one researcher (JL) according to Tier 2 protocol steps 7 – 10). Total counts for each morphology type identified from each photography method were compared to determine the percent accuracy of the low-resolution method. On average, larger sized microplastics had 99 % agreement between microscope counts and low-resolution photograph counts (SL 100 %, JL 91 %, JM 105 %) (Table S11). On average, 89 % of larger microplastic particles were consistently characterised in low-resolution photographs as they were under stereomicroscopy (Artificial turf 99 %, Foam 99 %, Pellets 86 %, Fragments 124 %, Film 94 %, Filament 85 %, Fishing line 38 %) (Fig. S5).

A final validation was performed by comparing data from a subset of 70 LRG filters, all processed and microplastics quantified based on: automated script-based counts (Tier 1), low-resolution photography-based characterisation (Tier 2), and high-resolution stereomicroscopy photography-based characterisation (Tier 2). Results from all three methods were compared using Mean Absolute Error (MAE), and coefficient of determination (R²) values calculated to evaluate the strength of the linear relationships between them. Strong positive correlations were observed between script and low-resolution photography counts (R² = 0.86, MAE = 1.90), low-resolution photography and stereomicroscopy counts (R² = 0.94, MAE = 1.77), and script and stereomicroscopy counts (R² = 0.81, MAE = 2.81). Low-resolution photography-based characterisation accurately counted microplastics within ±2 particles 91 % of the time, whereas the script counts within ±2 particles 81.4 % of the time. The low-resolution photography and script counts often agreed closely but still differed in about 20 % of cases. However, when the total microplastic count was fewer than 30 particles per sample, the script and low-resolution photography methods consistently returned values within ±2 particles of each other. Low-resolution photography-based characterisation (Tier 2), and high-resolution stereomicroscopy photography-based characterisation was compared. On average, 81 % of larger microplastic particles were consistently characterised in low-resolution photographs as they were under stereomicroscopy. Low-resolution photography-based characterisation of microplastic particles varied with morphology (Fig. S6). Mischaracterisation of photos is dependent on particle morphology (Artificial turf 80 %, Foam 110 %, Pellets 80 %, Fragments 115 %, Film 70 %, Filament 50 %, Fishing line 65 %).

### Microplastics chemical integrity post sample processing

To assess whether the sample processing protocol altered the chemical integrity of microplastics, a quality analysis of polymer spectra was conducted using FTIR, following the approach described by Santana et al. [20]. This validation was performed on five of the seven spiked microplastic types; PET and PVC were excluded due to their unsuccessful recovery during the density separation step. For each of the five retained polymer types, three replicate spectra were collected after completing the full sample processing workflow (*n* = 15 total). These treated spectra were compared to their corresponding untreated (original) spectra and to reference spectra from the NICODOM Polymers All Package library.

Spectral comparison was conducted using the *Compare* and *Search* functions in the PerkinElmer Spectrum IR software. The *Compare* function was applied to evaluate the similarity between treated and original spectra, detecting any changes in the position, shape, and intensity of key absorption peaks to highlight any structural alterations. The resulting correlation score quantified the degree of similarity, with values above 0.9 (90 %) indicating only minor or negligible changes attributable to sample processing.

To evaluate whether spectral changes introduced during sample processing could influence polymer identification, the *Search* function in the PerkinElmer Spectrum IR software was used to match the spectra of processed (treated) microplastics against (1) their corresponding untreated spectra and (2) the NICODOM Polymers All Package reference library. This step simulated the identification process used for environmental samples and assessed whether any spectral variations observed in the *Compare* analysis would affect the original classification of polymers. The primary criterion was that each microplastic retained its original polymer classification (e.g., polyethylene remained identified as polyethylene), thereby confirming that the processing protocol does not compromise the accuracy of polymer identification.

Results from the *Compare* analysis indicated that all treated microplastics (*n* = 15) achieved correlation scores equal to or greater than 0.9, meeting the threshold for spectral similarity (Table S12). Additionally, the *Search* analysis matched all treated microplastics to their original polymer type (Table S13). No notable spectral changes, such as the appearance of new peaks or alterations to existing peaks, were observed in the treated microplastics (Figure S7).

## Limitations

### Field collection

The specifically designed manta net only surveys the top 15 cm of the water column and, therefore, is only capturing microplastics on the surface of estuarine waters. As such, microplastics such as PVC and PET that have higher densities than estuarine waters (nominally between ∼1.000 to 1.022 g/cm³) are less likely to be collected using this technique; they are expected to be vertically transported through the water column to the sediment. Furthermore, the net mesh size is 0.3 mm, meaning this sampling technique can consistently collect microplastics greater than 0.3 mm, with smaller items being only opportunistically sampled and thus inherently underestimated.

### Sample processing

The alkaline digestion reagent 30 % KOH is effective in digesting organic matter such as lipids, and some carbohydrates and proteins present in the estuarine samples. However, it is less effective in digesting materials with highly stable or complex structures like cellulose, lignin, keratin, and chitin. This is an important consideration as these materials may remain in the sample after KOH digestion and generate interference that reduces the accuracy of the Rapid Count method, leading to an overestimation of counts. Additionally, the concentration of KOH used may potentially degrade some microplastic particles causing fragmentation however, no such effects were observed during method validation in this study.

Post digestion, the sample contents are treated with a saturated saline (NaCl) solution. This step further separates microplastics from denser organic matter, however, microplastics with a density greater than the NaCl solution (i.e., 1.202 g/cm³) will sink, and, therefore, not be recovered during filtration. Yet, as mentioned above, denser microplastics are not expected to predominate at the surface of estuarine systems, minimising the impact of this limitation.

### Tier 1: the rapid count method

The staining of microplastics with NR stain enables a rapid count estimate of microplastics in estuarine samples, however, Stanton et al., [15] concluded that NR staining underestimates certain polymers (i.e., polyester) and certain coloured polymers (i.e., black and brown), hence underestimates overall counts. Other limitations include:

• The inability to characterise microplastics by polymer.
• Generating an approximate but imprecise microplastics count.
• The inability of certain polymers, e.g., weathered ‘monocarboxy terminated’ polystyrene, artificial grass, and dark pigmented polymers, to adsorb the NR stain.
• The staining of residual biotic material, if not fully digested, e.g., carapace of plankton or cellulosic items, returning false ‘plastic’ positives. However, Meyers et al., [25] have developed machine learning techniques to distinguish biota from microplastics, as well as identify NR stained microplastics with an accuracy rate of 80 %.

Despite these limitations, NR staining can be effectively used for counting microplastics in broadscale assessments when these factors are accounted for. Furthermore, an automated script for fluorescent particle counting eliminates observer bias, ensuring standardised results. The accuracy of counts can be further improved by:

• Rinsing the sample filter disc liberally post NR staining to remove any residual NR stain.
• Ensuring the sample filter disc and microplastics are fully dry before photographing.
• Ensuring the light source is emitting at full strength. For battery powered light sources the battery must be fully charged, as the torch may emit less light as the power drains, in turn reducing florescence intensity.
• Ensuring an even coverage of the microplastics on the filter disc (<30 LRG, <40 MED, <100 SML) to avoid clumping, as this will limit script differentiation.
• Photographing samples against a black background to achieve high contrast and adjusting the angle of the blue light source to reduce reflection from the petri dish or filter disc.

Findings here support previous work [25] and demonstrate the Rapid Count method is a cost-effective (refer to Tables S1 -S4) and broadly applicable as a microplastics monitoring tool, sufficient for identifying hotspots of microplastics contamination (i.e., high loads), which can then be further investigated within the TMW to physically and chemically confirm the nature of the polymers.

### Tier 2: physical characterisation

Visual assessment techniques used to physically characterise microplastics can often be a source of observer bias, i.e., different observers may have varying levels of expertise and different thresholds for what constitutes a microplastic particle, or subjective assumptions for colour and shape. Further to this, there can be inconsistencies in classification, with some particles being overlooked (e.g., transparent film) and/or misidentified. To overcome this, well defined categories should be consistently applied [20,21]. Visually assessing microplastics is also labour intensive and increases in difficulty as the size of the items decrease. Analysis time can be further prolonged when visually analysing large quantities or complex samples, and over long periods, fatigue can impact accuracy. Additionally, using photographs to visually assess microplastics is challenging for some morphologies, as microscopic assessment allows for the inclusion of texture in characterisation of particles which is not possible in photographs. Filaments were often hard to visualise in low-resolution photographs and/or covered by larger items.

### Tier 3: chemical confirmation

Although FTIR instruments are evolving, standard FTIR instruments require an initial visual assessment to guide the selection of items for chemical analysis. As above, this introduces human error. Often this type of analysis requires the manual transfer of putative microplastics from the filter disc to the instrument which is not only time-consuming but also increases the risk of losing samples. Additionally, some microplastics are a complex mixture of polymers and additives (i.e., plasticisers, flame retardants, UV stabilisers), the latter potentially interfering in the polymer assignment; this is somewhat dependent on the spectral reference library against which the item is searched and the level of expertise in identifying diagnostic chemical signatures. Furthermore, FTIR instrumentation is not universally accessible (i.e., local and regional councils) and outsourcing this analysis can be expensive.

### Concentration calculations

Microplastic studies commonly report results as number of microplastics per sample, e.g., total number of microplastics per cubic meter of water (MP/m^3^). However, concentrations of other contaminants found in the environment are measured as amount of chemical dissolved in a solution, e.g., mass dissolved per volume (g/L). The suggestion for all microplastic water samples to be calculated as MP/m^3^ is still not aligned with other contaminants, although, as for asbestos, this will produce comparable data within the microplastics research community. To address the discrepancies with other contaminants, and supplement count data, it is recommended the filter disc be weighed pre and post filtering, to give an estimate weight of total microplastic contamination, noting that any non-plastic particles remaining on the filter post sample processing may also be included in the weight.

## Conclusions

A Tiered Microplastics Workflow (TMW), designed to enhance the accuracy and reliability of microplastic estimates in estuarine surface water samples, was developed and validated, offering multiple levels of analytical resolution. This workflow integrates proven methods, enabling researchers, industries, governments, and citizen scientists to tailor environmental monitoring initiatives to their specific needs. By accommodating varying resolutions of analysis, the TMW supports global comparisons of microplastics data, ensuring consistency and adaptability across different study scales and research questions.

The key findings are:

• **Field Collection:** Five-minute tows with a scaled-down manta net allows for the assessment of different estuarine waterways using different sized vessels, while effectively and efficiently sampling microplastics to assess spatial and temporal variation.
• **Sample processing:** Estuarine water samples require a stepwise processing protocol to improve sample clarification and microplastic detection. Applying a 30 % KOH digestion followed by NaCl density separation with vacuum degassing maximised processing efficiency.
• **The Rapid Count Method:** Nile Red staining in combination with ‘Stat-on-pixels’ (python script) provides an automated, cost-effective, and standardised way to count microplastics in high load samples. This method is suitable for large-scale abundance estimates where a 20 % margin of error with respect to microplastics density is tolerable and lack of information regarding the polymer is acceptable for the research question.
• **Data Harmonisation:** Calculating microplastic concentration (or abundance) at each collection site (MP/m^3^) allows for comparable datasets.
• **Application:** The Rapid Count method and TMW is currently being used by the NSW Department of Climate Change, Energy, the Environment & Water to establish a baseline for microplastic abundance in 120 NSW waterways, with the aim to develop a state-wide microplastic contamination grading system, i.e., Microplastics Report Cards.

Overall, the TMW enhances efficiency and allows for scalability and standardisation for robust microplastics monitoring on a broad scale.

## Declaration of competing interest

The authors declare that they have no known competing financial interests or personal relationships that could have appeared to influence the work reported in this paper.

## Data Availability

Data will be made available on request.

## Appendix Supplementary materials

Image, application 1

## References

[1] Nabi I., Bacha A.-U.-R., Zhang L. (2022). A review on microplastics separation techniques from environmental media. J. Clean. Prod..

[2] Halfar J. (2021). Disparities in methods used to determine microplastics in the aquatic environment: a review of legislation, sampling process and instrumental analysis. Int. J. Environ. Res. Public Health.

[3] Wootton N., Przeslawski R F.S. (2024). Field Manuals to Monitor Australian Waters.

[4] Miller M.E., Kroon F.J., Motti C.A. (2017). Recovering microplastics from marine samples: a review of current practices. Mar. Pollut. Bull..

[5] Malli A. (2022). Transport mechanisms and fate of microplastics in estuarine compartments: a review. Mar. Pollut. Bull..

[6] Grillo J.F. (2025). Rural village as a source of microplastic pollution in a riverine and marine ecosystem of the southern Venezuelan Caribbean. J. Contam. Hydrol..

[7] Przeslawski R. (2019). A suite of field manuals for marine sampling to monitor Australian waters. Front. Mar. Sci..

[8] Dehaut A. (2016). Microplastics in seafood: benchmark protocol for their extraction and characterization. Environ. Pollut..

[9] Prata J.C. (2019). Identifying a quick and efficient method of removing organic matter without damaging microplastic samples. Sci. Total. Environ..

[10] Thiele C.J., Hudson M.D., Russell A.E. (2019). Evaluation of existing methods to extract microplastics from bivalve tissue: adapted KOH digestion protocol improves filtration at single-digit pore size. Mar. Pollut. Bull..

[11] Thomas D. (2020). Sample preparation techniques for the analysis of microplastics in soil—A review. Sustainability..

[12] Shruti V.C. (2022). Analyzing microplastics with Nile Red: emerging trends, challenges, and prospects. J. Hazard. Mater..

[13] Shim W.J. (2016). Identification and quantification of microplastics using Nile Red staining. Mar. Pollut. Bull..

[14] Maes T. (2017). A rapid-screening approach to detect and quantify microplastics based on fluorescent tagging with Nile Red. Sci. Rep..

[15] Stanton T. (2019). Exploring the efficacy of Nile red in microplastic quantification: a costaining approach. Environ. Sci. Technol. Lett..

[16] Kunz A., Siña M. Microplastic extraction from beach sediments [Internet]. YouTube; 2021 [cited 2025 May 27]. Available from: https://www.youtube.com/watch?v=W8mLqxpzW0o.

[17] Schlawinsky M. (2022). Improved microplastic processing from complex biological samples using a customized vacuum filtration apparatus. Limnol. Oceanogr..

[18] Kroon F. (2018). A workflow for improving estimates of microplastic contamination in marine waters: a case study from North-Western Australia. Environ. Pollut..

[19] Wootton N. (2024). Research priorities` on microplastics in marine and coastal environments: an Australian perspective to advance global action. Mar. Pollut. Bull..

[20] Santana M.F.M. (2022). An assessment workflow to recover microplastics from complex biological matrices. Mar. Pollut. Bull..

[21] EMODnet Chemistry. 2023. EMODnet Micro‑Litter Types. Version 2023‑07‑25. NERC Vocabulary Server (SeaDataNet, Collection H01). https://vocab.nerc.ac.uk/collection/H01/current/. Accessed February 17, 2025.

[22] Prata J.C. (2021). Contamination issues as a challenge in quality control and quality assurance in microplastics analytics. J. Hazard. Mater..

[23] Miller M.E. (2021). Efficacy of microplastic separation techniques on seawater samples: testing accuracy using high-density polyethylene. Biol. Bull..

[24] Alves R.S. (2025). How does the tidal cycle influence the estuarine dynamics of microplastics?. Mar. Pollut. Bull..

[25] Meyers N. (2022). Microplastic detection and identification by Nile red staining: towards a semi-automated, cost- and time-effective technique. Sci. Total. Environ..

