# Supplementary material for: An integrated, tiered microplastic workflow, supporting rapid broadscale detection options
Source: MethodsX. 2025 Aug 5;15:103536. doi: 10.1016/j.mex.2025.103536 (PMC12343864; doi:10.1016/j.mex.2025.103536)
Supplement: Supplementary file 1 [file mmc1.docx]

**Supplementary material**

*“An integrated, tiered microplastic workflow, supporting rapid broadscale detection options.”*

Table 1. Field sampling materials (price as of January 2025).

| **Item** | **Manufacturer** | **Cost (AUD unless specified)** |
| --- | --- | --- |
| Manta net (400 mm W x 300 mm H x 1500 mm L, 300 μm mesh size) | Allied Filter Fabrics | $450 (single unit) |
| Net frame & bridle | Allied filter fabrics | $200 |
| 2 floats | Recycled |  |
| 15-m rope | Grunt | $23 |
| Flow meter | Imbros | $984.50 |
| 200 mL cod end | Allied Filter Fabrics | Incl. |
| 1 x vinyl storage bag (to avoid damage to net & contamination) | Ede Shade Solutions | $275 |
| 1 x 500 mL Cospak jar per tow (rinsed 3 times with MilliQ water & prefilled 100 mL 96% ethanol) | 500 mL White high-density polyethylene (HDPE) Round Jar with 83 mm Screw Neck (Cospak) | $217.80 (180 in carton) S1.21 each |
| MilliQ water | In house |  |
| Stopwatch | In phone |  |
| Permanent marker | Sharpie | $2.50 |
| Duct tape | Bear | $8.03 |
| 290 mm X 375 mm  Zip Lock Clear Plastic Bags | OzPack | $0.18 each |
| Tablet (or phone) form  / Data sheet | Open Data Kit (online form)  Tablet | $6,000 USD/annually  $800 |


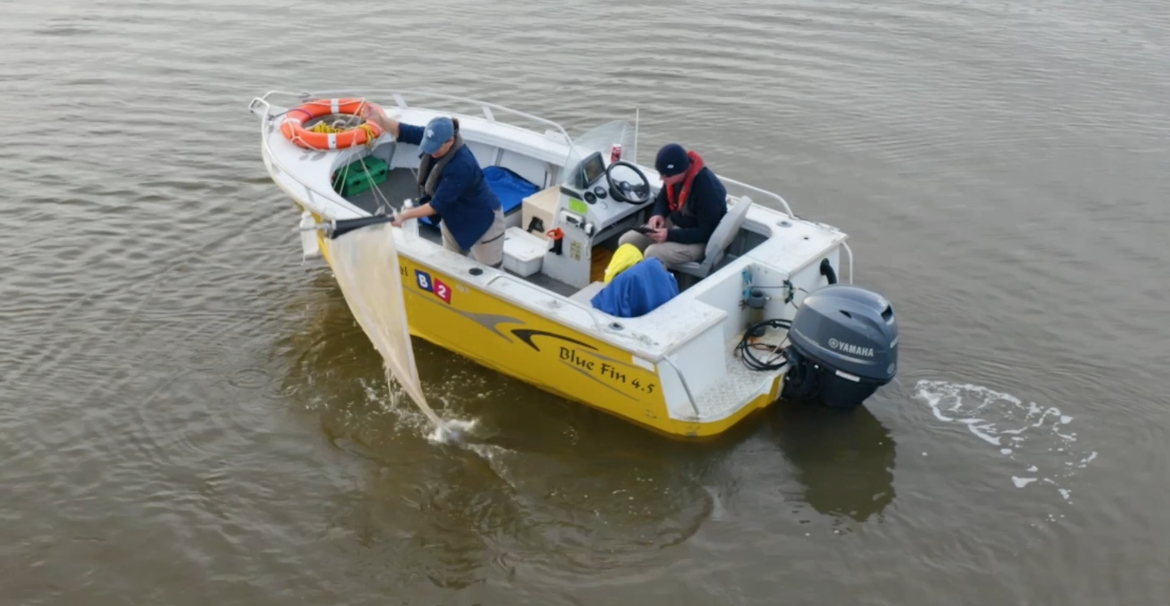


Fig. 1. Deploying the manta net starboard side of the vessel.


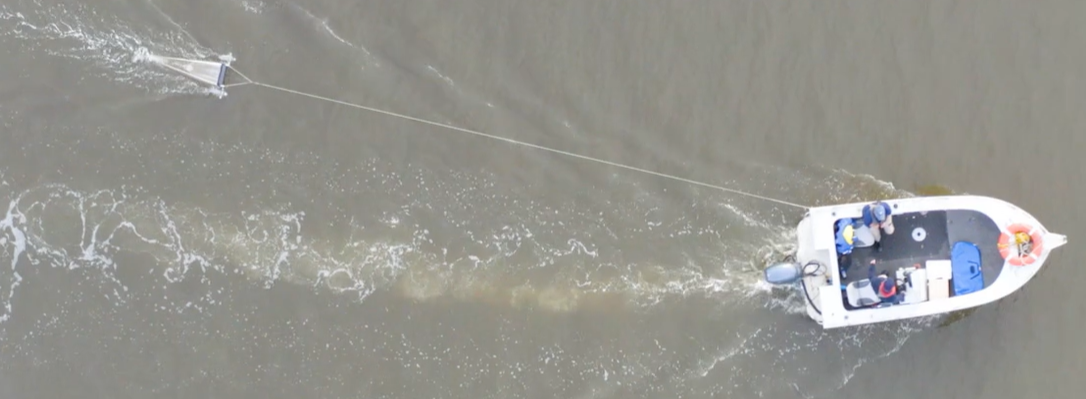


Fig. 2. Towing the manta net just outside the wake of the vessel.

Table 2. Sample processing materials (price as of January 2025).

| **Item** | **Manufacturer** | **Cost (AUD unless specified)** |
| --- | --- | --- |
| MilliQ water | In house |  |
| Labels | In house | $0.13 each |
| 5 mm and 0.2 mm sieve | In house |  |
| Reverse osmosis (RO) water | In house |  |
| 2 L glass beaker per sample | Livingstone (Low form, with spout, 130 mm D, 185 mm H, borosilicate glass) | $15.45 each |
| Potassium hydroxide (KOH)  214 g per sample | Sydney Solvents (4 kg) | $62 (4 kg),  $3.32 per sample |
| Stirring rod – glass | Livingstone stirring rod glass 7mm D X 400 mm L Round ends | $4.80 |
| Large glass petri dish (lid for beaker) | Anumbra Petri Dish, 150 Diameter x 25 Height mm, with Lid, Borosilicate Glass (Livingstone) | $7.30 |
| Oven (40°C) | In house |  |
| Safety goggles | Merck | $100 |
| Powder free nitrile gloves | Ni-Tek Nitrile Premium Gloves, AS NZ Standard Power free | $115 |
| Waste bucket |  | $10 |
| Acetic acid (CH₃COOH), Glacial 99.5% AR  (200 mL per sample) | Chem supply (West lab) | $48.40 (2.5 L),  $3.87 per sample |
| pH strips | MN (Australian Scientific) | $38.45/100 |
| 400 mL glass beaker per sample | Beaker Low Form, borosilicate (West lab) | $3.60 |
| Glass Petri dish (lid for 400 mL beaker) | Petri dish glass, 100 x 14 mm (West lab) | $6.30 |
| Sodium chloride (NaCl) 342 ppt | Chem supply (West lab) | $68.60 (5kg),  $4.70 per L |
| Squeeze bottles (1 for MilliQ water & 1 for NaCl solution) |  | $7.00 |
| Sonicator | In house |  |
| Vacuum Desiccator Chamber | West lab | $286 |
| Vacuum pump | HVACDIRECT  ROCKER 300 Oil-Free Vacuum Pump - 20 L/min | $669.30 |
| Tiered filtration system (Schlawinsky et al. 2022) | Built in house |  |
| Vacuum Filtration Manifold | Built in house | $5,000 |
| Stainless steel filters  Etching filter disc, Material: SS316L,  All - Diameter: 54 mm, Thickness: 0.2 mm, Pitch: 0.2 mm, Distance from hole to edge: 2 mm  Small - Hole size: 0.25 mm - Open area: 28%,  Medium - Hole size: 1 mm - Open area: 62%,  Large - Hole size: 2 mm - Open area: 74% | Boegger Industech Ltd | USD $1.90 ea |
| Forceps | Millipore stainless steel forceps, blunt end | $100 each |
| Nile Red stain (optional) | Merck | $152 (100 mg) |
| Acetone (optional) | Chem Supply Australia | $284 (4 L) |
| 55 mm glass petri dish  (3 per sample) | Livingstone (55 - 60 mm Diameter, sterile, with lid, recyclable polystyrene) | $3.40 (10 units per bag) |

Table 3. Materials required for the Rapid Count Method (price as of January 2025).

| **Item** | **Manufacturer** | **Cost (AUD unless specified)** |
| --- | --- | --- |
| Dark Room | In house |  |
| Digital Camera | Canon EOS 1500D | $799.95 |
| Lens | Canon MP-E 65 mm 1-5x f/2.8 Macro Lens | $1599 |
| Orange Filter |  | $56.95 |
| 2x Retort stand and clamp | In house |  |
| Blue light torch | Ultra Fire Blue Light Flashlight Hunting Torch 256 Yard 470 nm wavelength  Naccon 18650 Rechargeable Li-Ion Battery 2600 mAh 3.7 V  Single Channel Li-ion Battery Charger | $60.99  $16.95  $14.95 |
| Safety goggles | 3M SecureFit 400 Safety Glasses, Black/Brown frame, Scotchgard Anti-Fog, Orange Lens | $38 |
| SD card and reader | SanDisk Extreme PLUS 128 GB Verbatim Card Reader Hub | $29.95  $44 |
| Hard drive | ONE TOUCH Seagate | $208 |
| A computer with python installation | Relevant python modules: OpenCV | Free |
| [Python counting script](https://github.com/shivanesh/mp_counter) | NSW DCCEEW | Free |

Table 4. Materials used to physically characterise microplastics (price as of 2025).

| **Item** | **Manufacturer** | **Cost (AUD unless specified)** |
| --- | --- | --- |
| Stereomicroscope | Digitech 5MP USB Microscope | $259 |
| Digital Camera | Canon EOS 1500D | $799.95 |
| Flexible Clip-on White LED | Unbranded | $20 |
| [GIMP](https://www.gimp.org/)  [Image J software](https://imagej.nih.gov/ij) | GIMP  U.S. NIH, MD, USA | Free |
| Retort stand | Inhouse |  |
| SD card and reader | SanDisk Extreme PLUS 128 GB Verbatim Card Reader Hub | $29.95  $44 |
| Hard drive | ONE TOUCH Seagate | $208 |


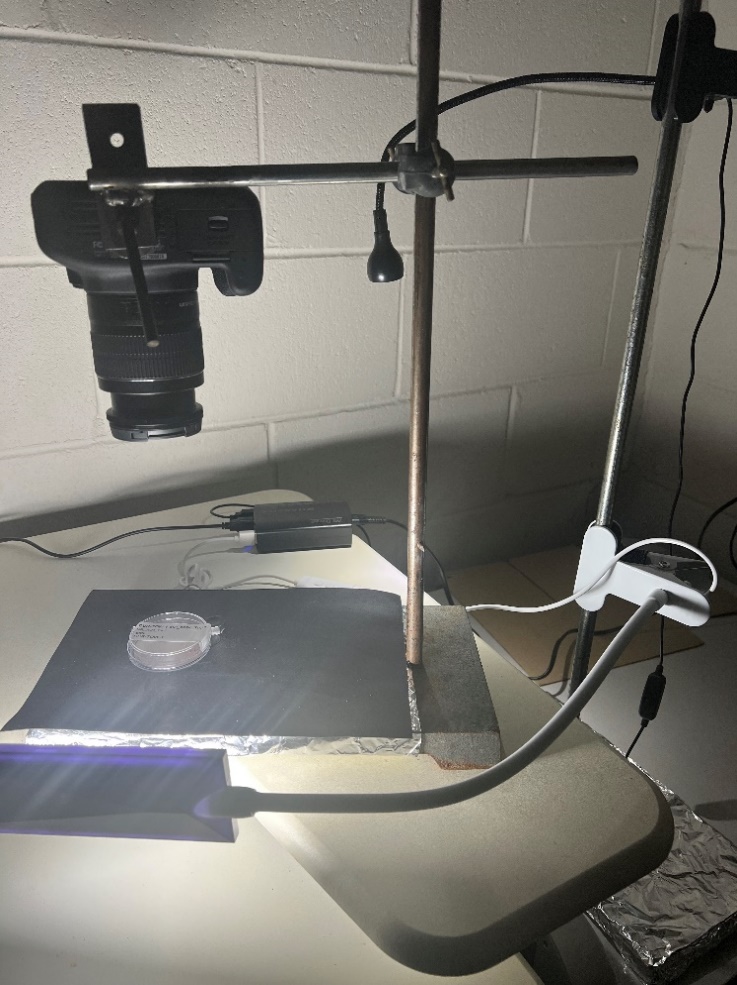


Fig. 3. Digital camera position optimised to capture images of microplastics under white light. The camera was mounted on a retort stand and positioned 18 cm from the fixed glass petri dish. The white light is positioned at a 45-degree angle to the camera and 10 cm from the petri dish.

Table 5. Colours of microplastics (adapted from Santana, Kroon [20]).

| **Name of colour** | **Shade of colour** |
| --- | --- |
| Blue |  |
| Blue-purple |  |
| Purple |  |
| Pink |  |
| Red |  |
| Orange |  |
| Yellow |  |
| Yellow-green |  |
| Green |  |
| Green-blue |  |
| Grey |  |
| Black |  |
| White |  |
| Transparency |  |

Table 6. Microplastic morphologies (adopted from [21]).

| **Label** | **Definition/Subcategory** | **Photograph** |
| --- | --- | --- |
| **Filament**  [H0100004](http://vocab.nerc.ac.uk/collection/H01/current/H0100004/) | Slender thread-like micro-litter particles. Red arrow pointing to a white filament. | 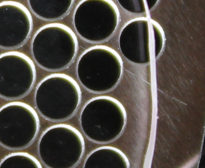 |
|  | Fishing line | 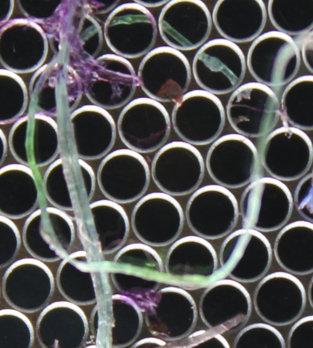 |
| **Film**  [H0100005](http://vocab.nerc.ac.uk/collection/H01/current/H0100005/) | Thin planar micro-litter particles, in which the thickness is small compared to the length and width. Films are flexible compared to more rigid Fragments. | 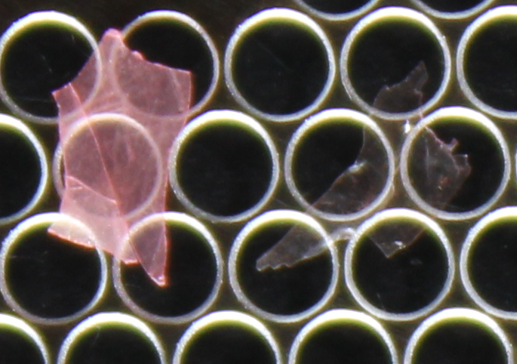 |
|  | Artificial Grass. | 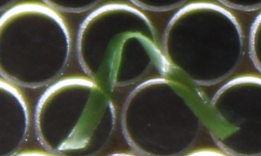  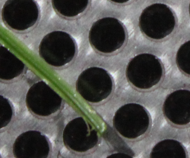 |
| **Foam**  [H0100006](http://vocab.nerc.ac.uk/collection/H01/current/H0100006/) | Flexible micro-litter particles. Squishy. Can be sphere shaped, flat, or irregular shaped. | 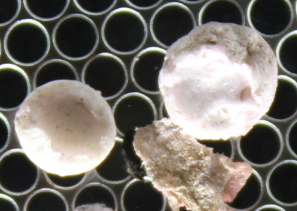 |
| **Fragment**  [H0100002](http://vocab.nerc.ac.uk/collection/H01/current/H0100002/) | Hard, irregularly shaped micro-litter particles with serrated jagged edges that may be rounded or angular. | 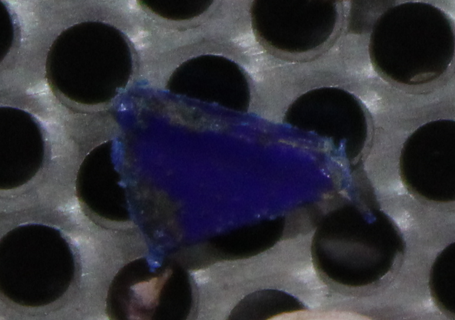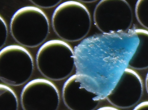 |
| **Non-Plastic** | A generic term for any kind of item that is naturally derived. Here, biological material from a crustacean. | 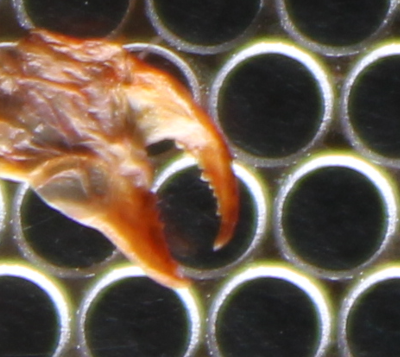 |
| **Pellets**  [H0100003](http://vocab.nerc.ac.uk/collection/H01/current/H0100003/) | Hard micro-litter particles from industrial origin only. Round or cylindrical in shape. | 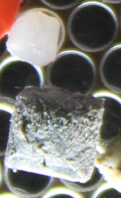 |
|  | Nurdles (plastic feed stock). | 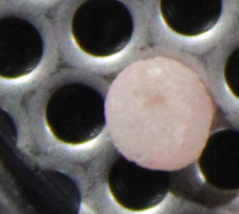 |
| **Rubber** | Rubber crumb (recycled tyres) | 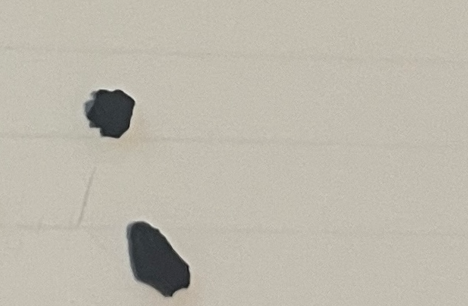 |

Table 7. Materials used to chemically characterise microplastics (price when purchased in 2021).

| **Item** | **Manufacturer** | **Cost (AUD unless specified)** |
| --- | --- | --- |
| FTIR iS50 benchtop ADV w/ATR  Model: 912A1098 | Thermo Fisher Scientific | Inhouse |
| Isopropanol | Sydney Solvents (5 L) | $26.00 |
| NICODOM IR Polymers All Package, 6701 spectra | <http://www.ir-spectra.com/> | 3,390 euros |

Table 8. Controls implemented to avoid extraneous microplastic contamination.

| **Control** | **Description** | **Image** |
| --- | --- | --- |
| **Vinyl Bag** | To avoid airborne contamination of the net, it was stowed and carried in the bag the entire time when not in use. | **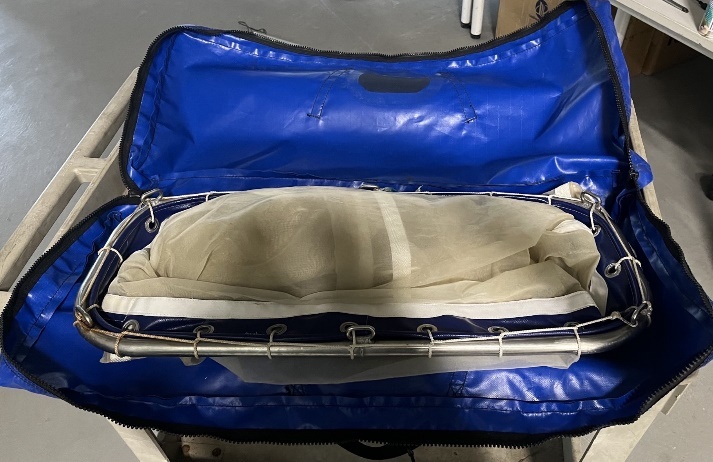** |
| **Net** | Before use, to avoid any airborne microplastic contamination, the manta net was rinsed by towing it in the water for a period of 1 minute without the cod end. | 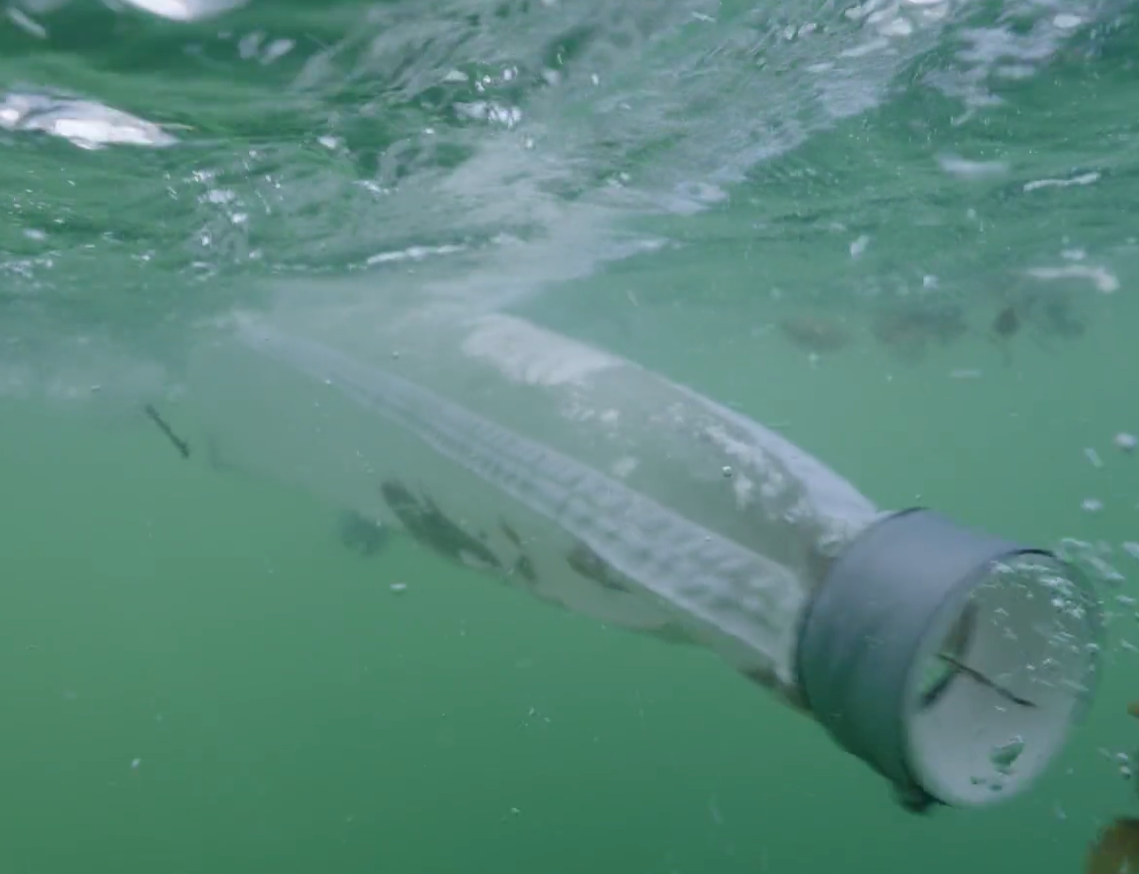 |
| **Cod End** | Before use, to avoid any airborne microplastic contamination, the cod end was triple rinsed with MilliQ water prior to net attachment. | 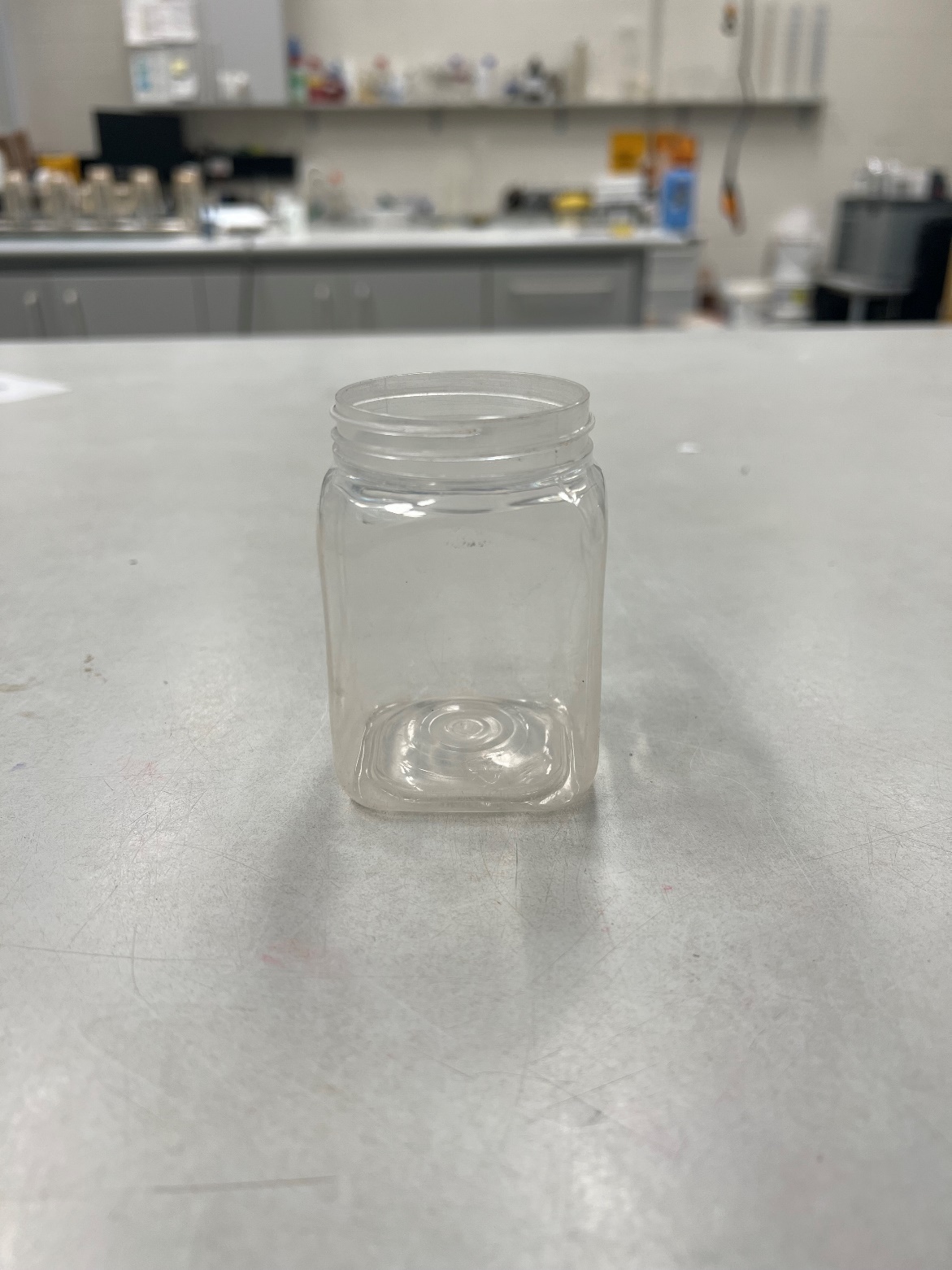 |
| **Sampling Jar** | Before use, to avoid any microplastic contamination, the sample jar was triple rinsed with MilliQ water in the laboratory and prefilled with 100 mL of 96% filtered <0.25 µm Ethanol. Sample jar was only opened in the field when sample was ready to be transferred. The jar was capped at all other times during storage. | 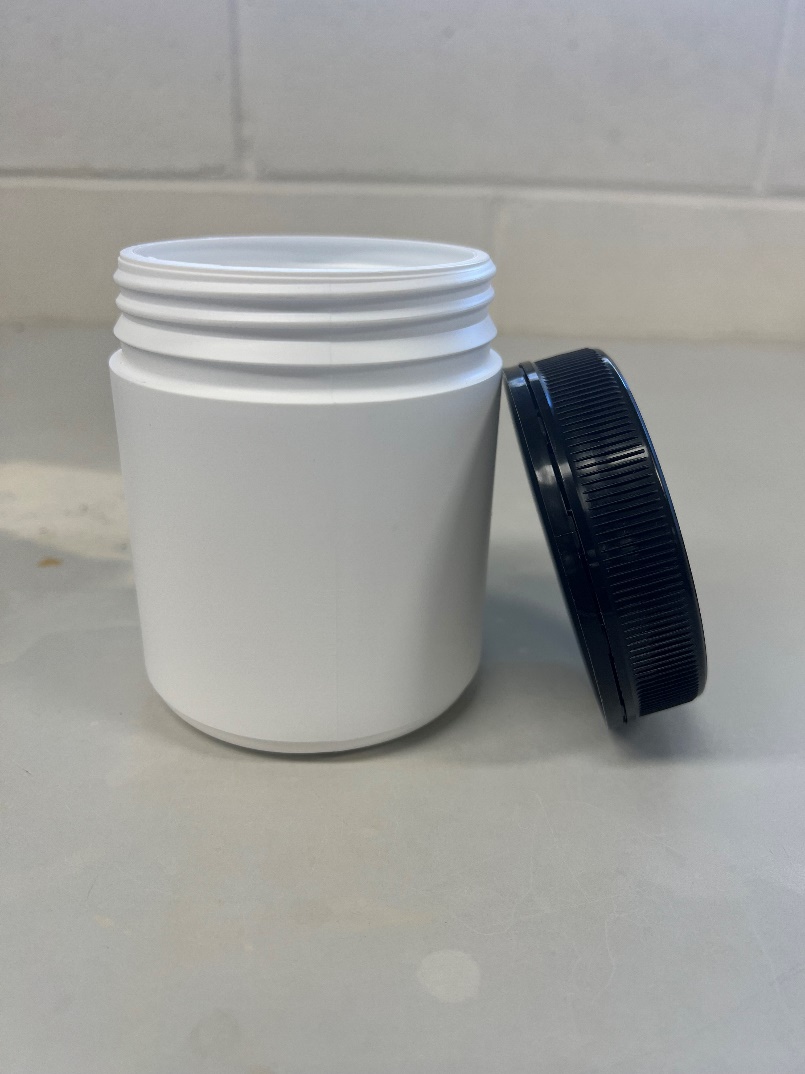 |
| **Lab Coat** | Brightly coloured cotton clothing and lab coats were worn so that any contamination can be readily identified. | 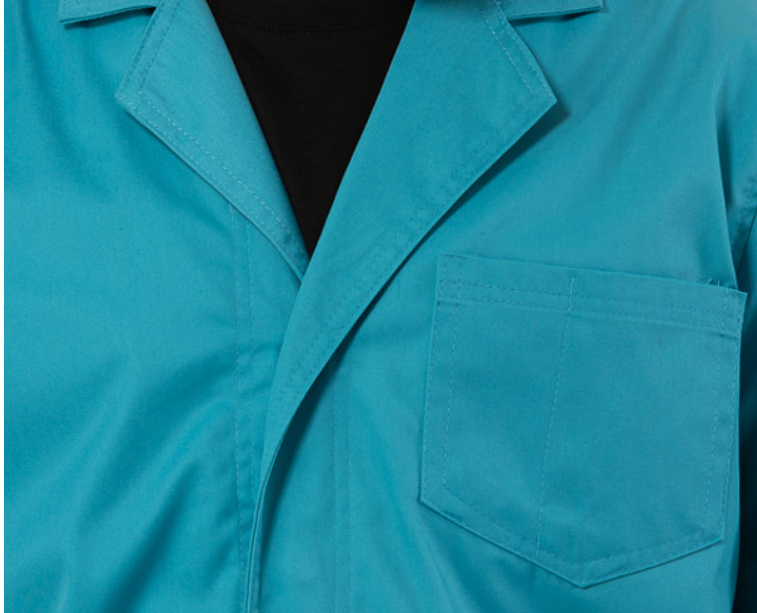 |
| **Laboratory equipment** (Beakers, filter discs, filtration system, forceps) | Before use, to avoid any microplastic contamination, all equipment was triple rinsed with MilliQ water. | 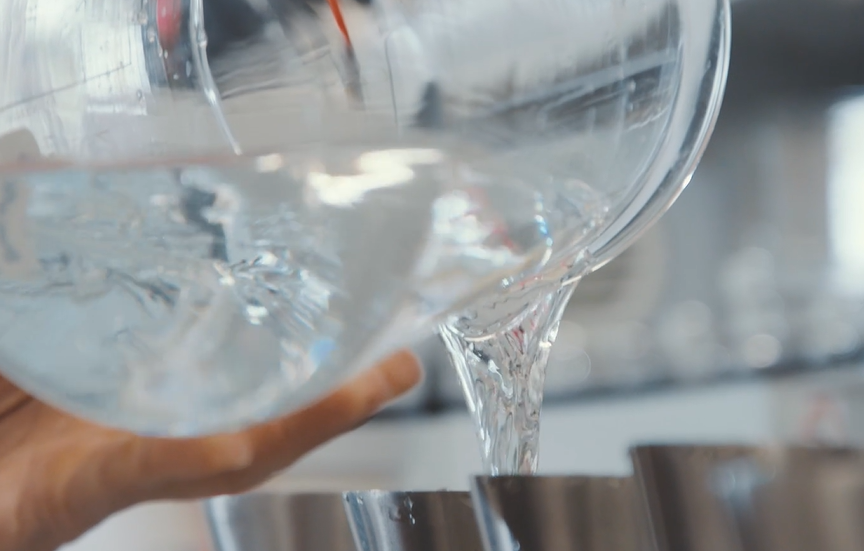 |
| **Glass Lids** | To avoid any microplastic contamination, glass lids (triple rinsed with MilliQ water) were placed on top of all beakers and only removed when sample was manipulated (e.g., addition of KOH or sample filtration). | 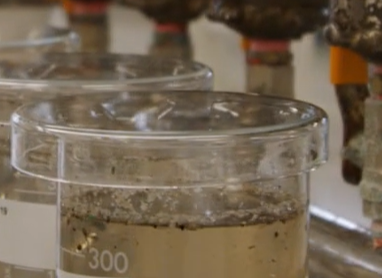 |
| **Filtration of reagents** | All chemical reagents used (NaCl, Ethanol, Nile Red solution) were filtered (<0.25 µm) prior to use. | 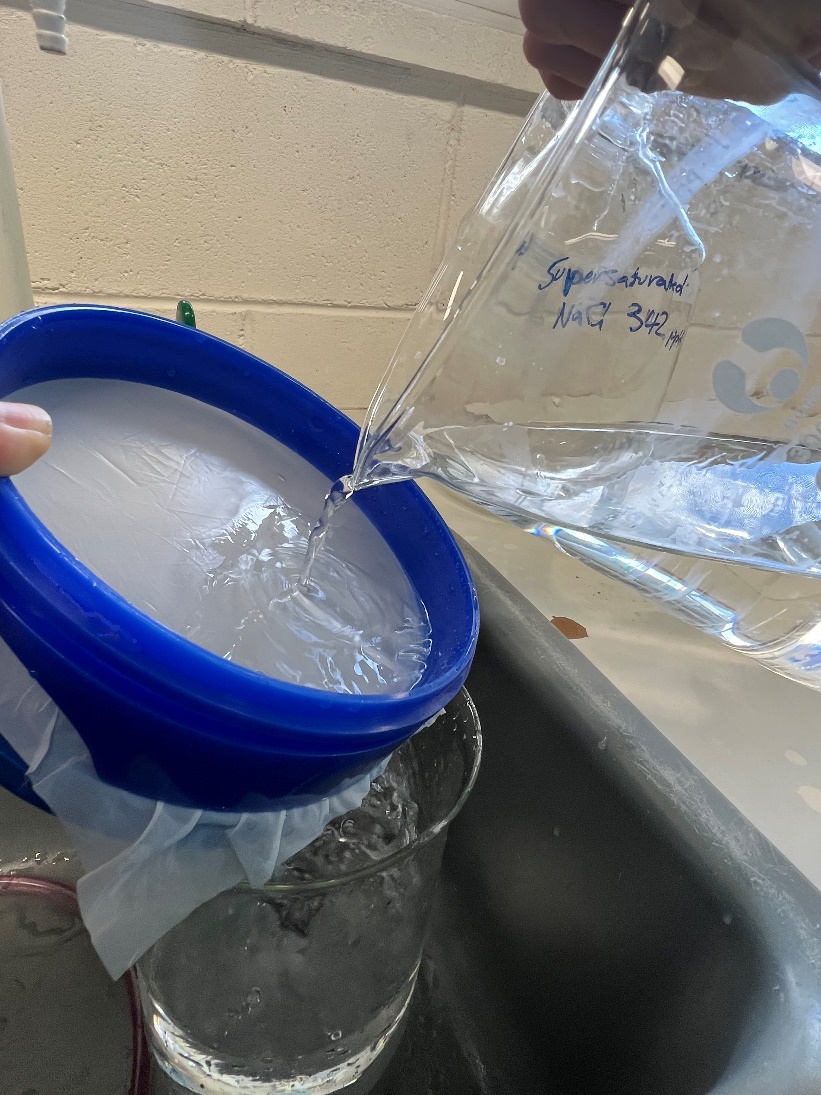 |
| **Contaminants library** | To identify extraneous contamination from unavoidable plastic gear, samples from items that were used during field collection (e.g., ropes, nets, duct tape, bags, vessel paint, and field teams clothing) were collected. These items were analysed and added to the FTIR contaminants library. Confirmed microplastics, based on FTIR, found in samples were analysed against this contaminant library and removed from the final dataset if spectra matches were above 90% and physical characteristics were also similar [18]. Note this was only conducted for the Stage 3 approach. | 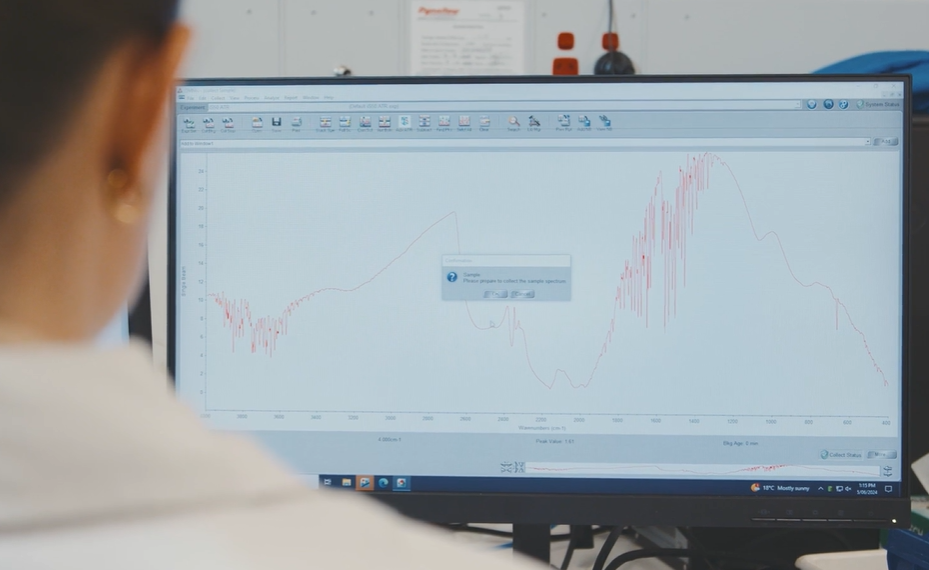 |

Table 9. Blanks and controls used throughout microplastic sampling and processing.

| **Type of blank** | **Description** |
| --- | --- |
| **Field blank** | Near the field crews, pre-cleaned jars were left open (lid unscrewed) whilst sampling was being undertaken, exposing the sample to the air. These samples were then processed following the same protocol used for the field samples to determine if any contamination had occurred. |
| **Laboratory blank** | Whilst undertaking the laboratory processing and analysis (i.e., from sieving to FTIR), a beaker with MilliQ water was always placed next to the technician with the lid removed. These samples were then processed following the same protocol used for the field samples to determine if any contamination had occurred. |
| **Procedural blank** | MilliQ water underwent the same treatment as the environmental samples, sieving, digestion, density separation, filtration, staining, photographing, and microscopy to determine if any contamination had occurred. |
| **Contaminant library** | Microplastics found in the three blank types (field, laboratory, procedural) were added to an in-house contaminant library and if found to match greater than 90% to an item and have identical physical characteristics, the sample item was removed from the final dataset (Kroon et al., 2018) [18].  Note the contaminant library is only used by the polymer identification method (Stage 3). |

Table 10. Microplastic recovery rates. Visual counts of microplastics were conducted after each step of the method, namely post sieving, post digestion with 30% KOH, and post filtration, and recorded along with the recovery percentage (%), and the recovery % of microplastics using the rapid method. The microplastics used to spike treatments included PS, LDPE, PE, PP, PVC, PET, EPDM (refer to Table 2 for definitions). Recovery rates (%) exclude PVC and PET due to their density being greater than NaCl and, therefore, sinking to the bottom of the beaker.

| **Spike Sample No** | **Initial count** | **Post sieve**  **count** | **Post 30% KOH**  **count** | **Post filtration**  **count** | **Total Loss**  **count** | **Recovery rate  (%)** | **Script  count** | **Accuracy of script count vs physical count (%)** |
| --- | --- | --- | --- | --- | --- | --- | --- | --- |
| 1 | 70 | 70 | 69 | 69 | -1 | 99% | 69 | 100% |
| 2 | 72 | 72 | 71 | 71 | -1 | 99% | 72 | 101% |
| 3 | 71 | 71 | 71 | 71 | 0 | 100% | 71 | 100% |
| 4 | 75 | 75 | 75 | 75 | 0 | 100% | 74 | 99% |
| 5 | 73 | 73 | 73 | 70 | -3 | 96% | 72 | 103% |
| 6 | 70 | 70 | 70 | 70 | 0 | 100% | 69 | 99% |


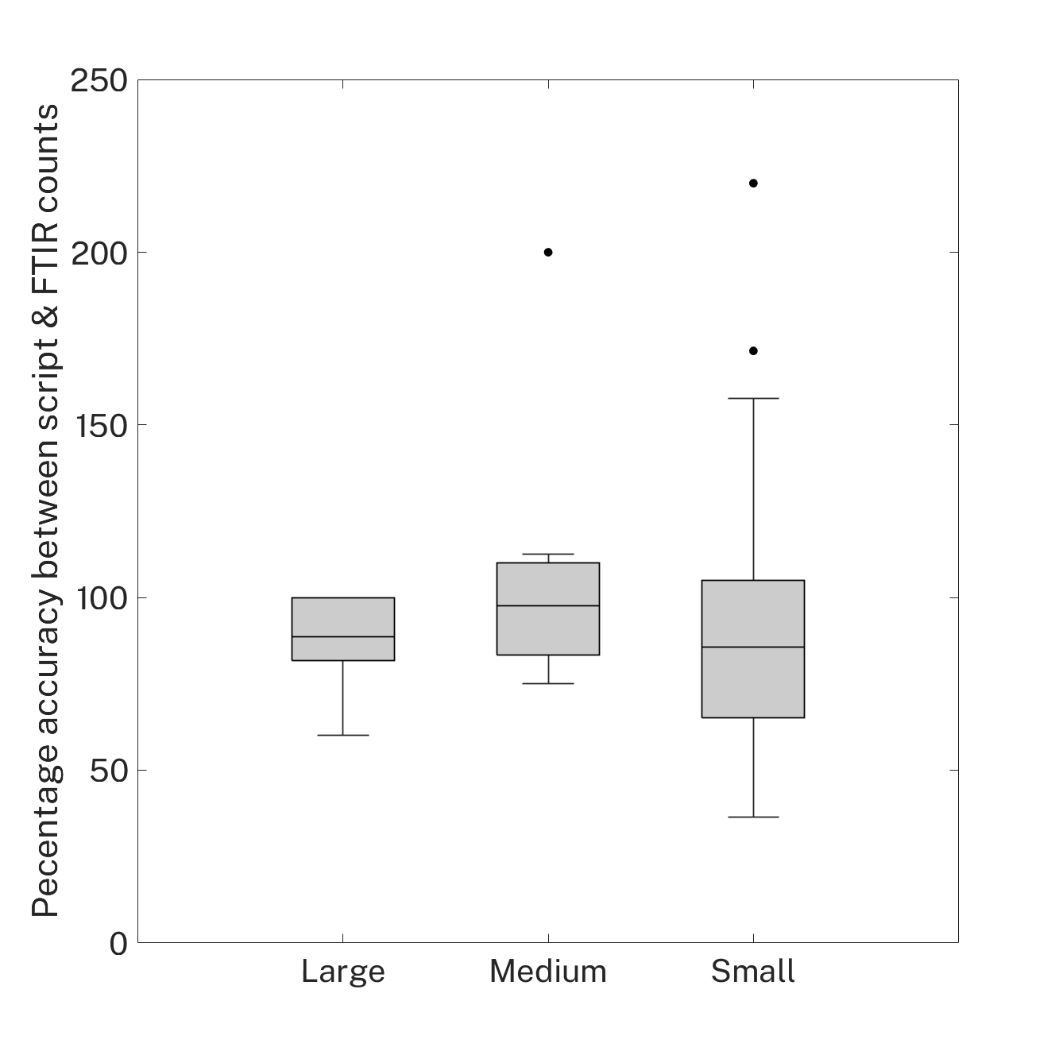


Fig. 4. Box and whiskers plot displaying the percentage accuracy range of the script counts when compared to the FTIR counts. 100% demonstrates that the script accurately counted 100% of the microplastics, as confirmed by FTIR. >100% indicates an overestimate and <100% an underestimate. Large (LRG) are items ranging between 2 – 5 mm in size; Medium (MED) 2 – 1 mm; and Small (SML) 1 – 0.25 mm.

Table 11. Photograph and microscopy microplastic morphology counts.

| CODE | TOTAL | ARTIFICIAL TURF | FOAM | PELLETS | FRAGMENT | FILM | FILAMENT | FISHING LINE | IDENTIFIER | ACCURACY % |
| --- | --- | --- | --- | --- | --- | --- | --- | --- | --- | --- |
| 1 | 14 | 1 | 10 |  | 3 |  |  |  | MICRO |  |
| 1 | 14 | 1 | 10 |  | 3 |  |  |  | SL | 100% |
| 1 | 14 | 1 | 10 |  | 3 |  |  |  | JL | 100% |
| 1 | 14 | 1 | 10 |  | 3 |  |  |  | JM | 100% |
| 2 | 16 | 1 | 11 |  | 1 |  |  | 3 | MICRO |  |
| 2 | 16 | 1 | 11 |  | 1 |  |  | 3 | SL | 100% |
| 2 | 16 | 1 | 11 |  | 4 |  |  |  | JL | 100% |
| 2 | 16 | 1 | 11 |  | 4 |  |  |  | JM | 100% |
| 3 | 18 | 2 | 10 |  | 5 |  |  | 1 | MICRO |  |
| 3 | 18 | 2 | 10 |  | 5 |  |  | 1 | SL | 100% |
| 3 | 18 | 2 | 10 |  | 6 |  |  |  | JL | 100% |
| 3 | 18 | 2 | 10 |  | 5 |  |  | 1 | JM | 100% |
| 4 | 89 | 11 | 30 | 3 | 25 | 10 | 9 | 1 | MICRO |  |
| 4 | 77 | 9 | 34 | 3 | 25 |  | 6 |  | SL | 87% |
| 4 | 72 | 8 | 26 | 2 | 27 | 9 |  |  | JL | 81% |
| 4 | 105 | 9 | 41 | 3 | 38 | 10 | 4 | 0 | JM | 118% |
| 5 | 106 | 1 | 71 | 2 | 22 | 10 |  |  | MICRO |  |
| 5 | 106 | 1 | 73 | 1 | 22 | 5 | 4 |  | SL | 100% |
| 5 | 80 | 1 | 46 | 2 | 22 | 9 |  |  | JL | 75% |
| 5 | 106 | 1 | 79 | 2 | 18 | 6 | 0 | 0 | JM | 100% |
| 6 | 56 | 1 | 34 | 1 | 15 | 5 |  |  | MICRO |  |
| 6 | 61 | 1 | 41 | 1 | 12 | 5 | 1 |  | SL | 109% |
| 6 | 45 | 2 | 23 | 1 | 10 | 9 |  |  | JL | 80% |
| 6 | 50 | 1 | 34 | 1 | 7 | 7 | 0 | 0 | JM | 89% |
| 7 | 62 | 10 | 5 |  | 27 | 20 |  |  | MICRO |  |
| 7 | 54 | 8 | 7 |  | 21 | 18 |  |  | SL | 87% |
| 7 | 50 | 8 |  |  | 32 | 10 |  |  | JL | 81% |
| 7 | 55 | 8 | 6 | 0 | 26 | 15 | 0 | 0 | JM | 89% |
| 8 | 29 |  | 11 | 3 | 12 | 3 |  |  | MICRO |  |
| 8 | 32 | 1 | 10 | 3 | 11 | 6 | 1 |  | SL | 110% |
| 8 | 28 |  | 9 | 3 | 13 | 3 |  |  | JL | 97% |
| 8 | 33 | 0 | 15 | 2 | 14 | 2 | 0 | 0 | JM | 114% |
| 9 | 14 |  | 1 | 1 | 7 |  | 1 | 4 | MICRO |  |
| 9 | 13 |  |  | 1 | 9 |  | 1 | 2 | SL | 93% |
| 9 | 11 |  | 1 |  | 7 |  | 1 | 2 | JL | 79% |
| 9 | 14 | 0 | 2 | 1 | 7 | 0 | 2 | 2 | JM | 100% |
| 10 | 7 |  | 2 |  | 4 | 1 |  |  | MICRO |  |
| 10 | 8 |  | 2 |  | 5 | 1 |  |  | SL | 114% |
| 10 | 8 |  | 2 |  | 5 | 1 |  |  | JL | 114% |
| 10 | 10 |  | 2 | 0 | 7 | 1 | 0 | 0 | JM | 143% |


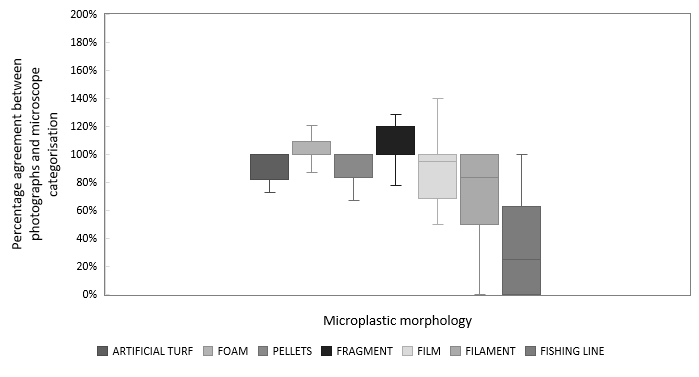


Fig. 5. Box and whiskers plot displaying the percentage accuracy range of the low-resolution photographs when compared to the microscope counts n=10. 100% demonstrates that the photos categorised 100% of the microplastics, categoriesed by the microscope. >100% indicates an overestimate and <100% an underestimate.


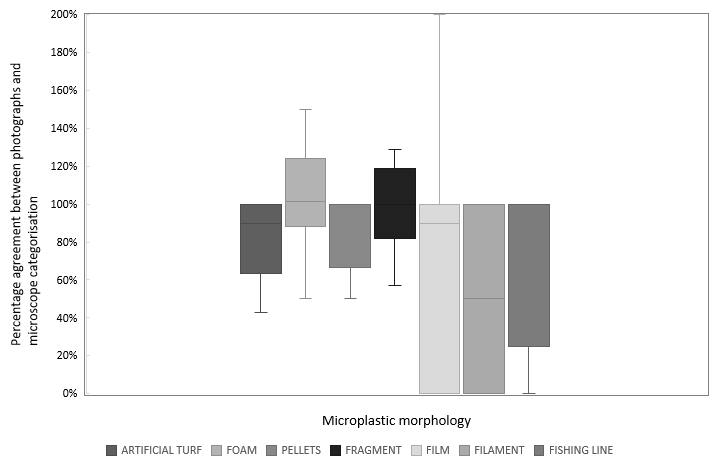


Fig. 6. Box and whiskers plot displaying the percentage accuracy range of the low-resolution photographs when compared to the microscope counts n=70. 100% demonstrates that the photos categorised 100% of the microplastics, categoriesed by the microscope. >100% indicates an overestimate and <100% an underestimate.

|  | *Please Select Subject Area from dropdown list* |
| --- | --- |

Table 12. Results from the *Comparison* analysis between the spectra of the spiked (i.e., treated) microplastics and their original untreated spectra.

| **Spiked Microplastic** | **Correlation with original untreated spectra** |
| --- | --- |
| PE (yellow) spike # 1 | 0.995479 |
| PE (yellow) spike # 2 | 0.99518 |
| PE (yellow) spike # 3 | 0.990864 |
| LDPE (green) spike # 1 | 0.924574 |
| LDPE (green) spike # 2 | 0.974825 |
| LDPE (green) spike # 3 | 0.945587 |
| PP (blue) spike # 1 | 0.947381 |
| PP (blue) spike # 2 | 0.988623 |
| PP (blue) spike # 3 | 0.982632 |
| PS (white) spike #1 | 0.919918 |
| PS (white) spike #2 | 0.978341 |
| PS (white) spike #3 | 0.931799 |
| EPDM (red) spike #1 | 0.99562 |
| EPDM (red) spike #2 | 0.974467 |
| EPDM (red) spike #3 | 0.949956 |

Table 13. Results from the FTIR (Spectrum IR) spectra *Search* of spiked microplastics (treated) against (1) their untreated original spectra and (2) the NICODOM Polymers All Package reference library.

1. **Untreated original spectra**

| **Spiked microplastic** | **Search reference** | **Library match score** |
| --- | --- | --- |
| PE (yellow) spike # 1 | PE (yellow) original | 0.99695 |
| PE (yellow) spike # 2 | PE (yellow) original | 0.996 |
| PE (yellow) spike # 3 | PE (yellow) original | 0.992288 |
| PE (green) spike # 1 | PE (green) original | 0.996154 |
| PE (green) spike # 2 | PE (green) original | 0.996798 |
| PE (green) spike # 3 | PE (green) original | 0.992949 |
| PP (blue) spike # 1 | PP (blue) original | 0.994289 |
| PP (blue) spike # 2 | PP (blue) original | 0.997259 |
| PP (blue) spike # 3 | PP (blue) original | 0.998901 |
| PS (white) spike #1 | PS (white) original | 0.989305 |
| PS (white) spike #2 | PS (white) original | 0.979556 |
| PS (white) spike #3 | PS (white) original | 0.998041 |
| EPDM (red) spike #1 | EPDM (red) original | 0.995974 |
| EPDM (red) spike #2 | EPDM (red) original | 0.987937 |
| EPDM (red) spike #3 | EPDM (red) original | 0.965824 |

1. **NICODOM Polymers All Package reference library**

| **Spiked microplastic** | **Search reference** | **Library match score** |
| --- | --- | --- |
| PE (yellow) spike # 1 | PAD0002.SPC POLYETHYLENE LINEAR LOW DENSITY, ESCORENE LLN100 4YB;9002-8 | 0.992136 |
| PE (yellow) spike # 2 | PAD0144.SPC POLYETHYLENE PLASTICIZED #1;9002-88-4;(C2H4)N,WWW.IR-SPECTR | 0.990817 |
| PE (yellow) spike # 3 | PAD0144.SPC POLYETHYLENE PLASTICIZED #1;9002-88-4;(C2H4)N,WWW.IR-SPECTR | 0.9924 |
| PE (green) spike # 1 | PAD0002.SPC POLYETHYLENE LINEAR LOW DENSITY, ESCORENE LLN100 4YB;9002-8 | 0.986741 |
| PE (green) spike # 2 | PAD0002.SPC POLYETHYLENE LINEAR LOW DENSITY, ESCORENE LLN100 4YB;9002-8 | 0.986307 |
| PE (green) spike # 3 | PEC0338.SPC POLYETHYLENE LINEAR LOW DENSITY, ESCORENE LLN100 4YB, 9002- | 0.988073 |
| PP (blue) spike # 1 | FB230.SP FB230, INNOVA, OLEFIN PP, AMERICAN FIBERS, COPYRIGHT NICODOM 2 | 0.98392 |
| PP (blue) spike # 2 | FB230.SP FB230, INNOVA, OLEFIN PP, AMERICAN FIBERS, COPYRIGHT NICODOM 2 | 0.98574 |
| PP (blue) spike # 3 | FB230.SP FB230, INNOVA, OLEFIN PP, AMERICAN FIBERS, COPYRIGHT NICODOM 2 | 0.98662 |
| PS (white) spike #1 | F00946.SP F00946POLYSTYRENE STANDARD 20'000 CERTIFIED ACC. TO DIN | 0.991358 |
| PS (white) spike #2 | PAD0348.SPC POLY(STYRENE::BUTADIENE::STYRENE);9003-55-8;(C4H6)X(C8H8)N, | 0.975116 |
| PS (white) spike #3 | PAD0207.SPC  POLYSTYRENE,MONOCARBOXY TERMINATED;9003-70-7,9003-53-6;(C8 | 0.97898 |
| EPDM (red) spike #1 | PAD0364.SPC POLY(ETHYLENE:PROPYLENE:DIENE) (50% ETHYLENE, 4% DIENE);250 | 0.950806 |
| EPDM (red) spike #2 | PAD0355.SPC DUTRAL PMX 9705, COPOLYMER EPDM TYPE;25038-36-2;NICODOM,POL | 0.956977 |
| EPDM (red) spike #3 | PAD0355.SPC DUTRAL PMX 9705, COPOLYMER EPDM TYPE;25038-36-2;NICODOM,POL | 0.970942 |

*
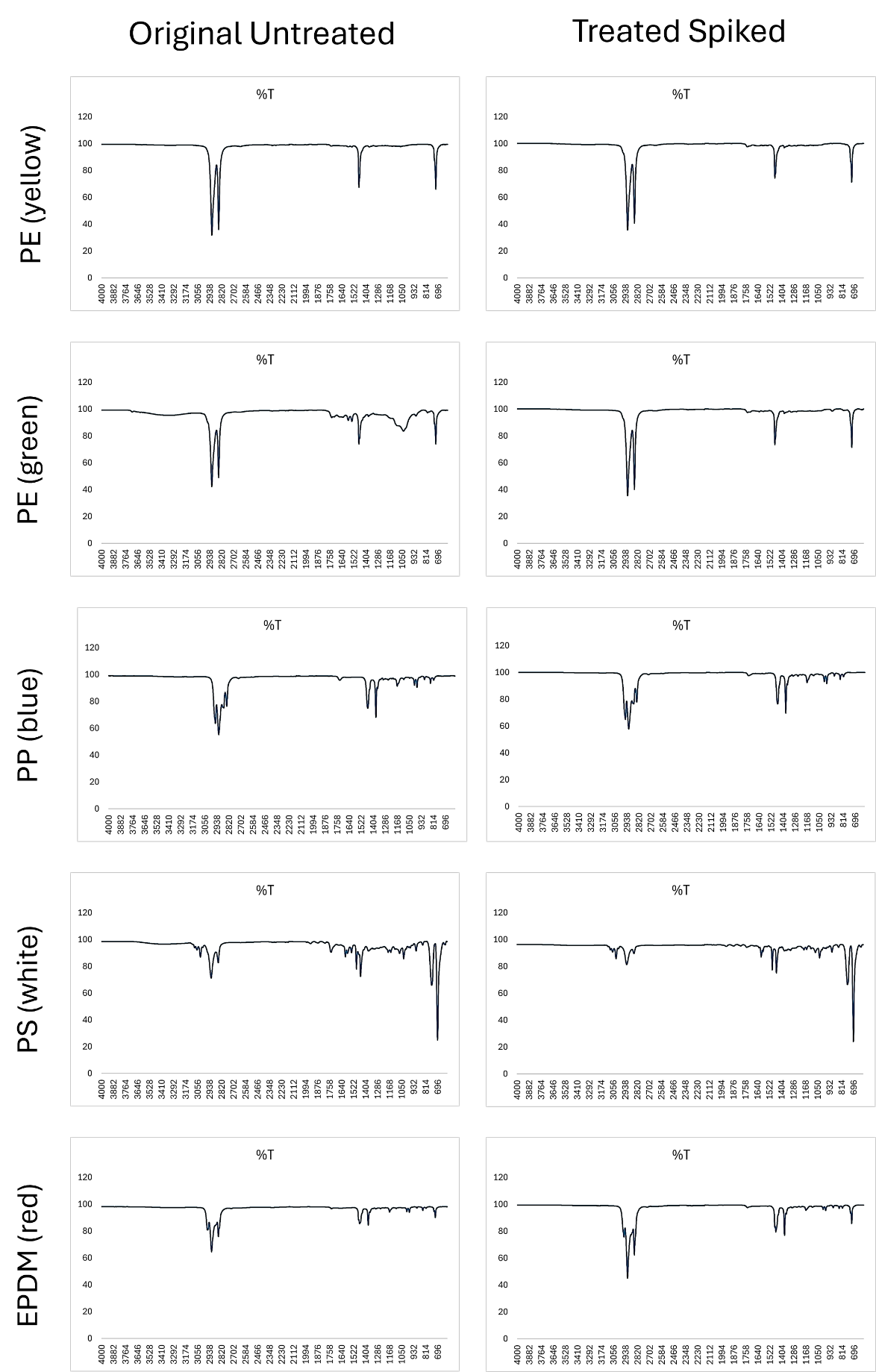
*

Fig. 7. Spectra of spiked microplastics (PE yellow and green, PP blue, PS white and EPDM red) before (original untreated) and after (spiked treated) microplastics separation workflow.
